# Redox‐Acidity Interplay in Eu‐Promoted PtSn_2_ Catalysts for Selective and Stable Propane Dehydrogenation

**DOI:** 10.1002/anie.202512853

**Published:** 2025-10-23

**Authors:** María I. Valls, Jesús Ara, Sonia Escolástico, Sonia Remiro‐Buenamañana, David Catalán‐Martínez, Julien Grand, Moritz Kindelmann, Joachim Mayer, Simona Somacescu, Daniel Curulla‐Ferré, Jose M. Serra

**Affiliations:** ^1^ Instituto de Tecnología Química Consejo Superior de Investigaciones Científicas Universitat Politècnica de València Valencia 46022 Spain; ^2^ TotalEnergies S.E, Zone Industrielle Feluy C Seneffe B7181 Belgium; ^3^ Ernst Ruska‐Center for Microscopy and Spectroscopy with Electrons (ER‐C) Forschungszentrum Jülich GmbH 52425 Juelich Germany; ^4^ Central Facility for Electron Microscopy (GFE) RWTH Aachen University 52074 Aachen Germany; ^5^ “Ilie Murgulescu” Institute of Physical Chemistry Romanian Academy Spl. Independentei 202 Bucharest 060021 Romania

**Keywords:** Acidity, Catalyst, Europium, Platinum, Propane dehydrogenation, Redox catalyst

## Abstract

Heterogeneous catalysts based on Pt alloys are widely employed in propane dehydrogenation (PDH), yet challenges such as coking and poor nanoparticle stability hinder their broader industrial deployment. Strategies to enhance dispersion and tune the catalyst surface properties remain at the forefront of catalyst design. Here, we demonstrate a new class of PtSn_2_‐based catalysts promoted by rare‐earth elements for efficient and stable PDH. Among the rare‐earth screened, europium (Eu) delivers the most pronounced promotional effect, enabling the formation of ∼1.3 nm PtSn_2_ nanoparticles with improved thermal stability. Through its redox flexibility (Eu^3+^/Eu^2+^), Eu modulates the electronic environment of Pt, tunes surface acidity, and suppresses coke accumulation by directing carbon species away from active sites and onto the support. This work shows that rare‐earth elements can serve as multifunctional promoters in alloy catalysts, influencing both structural dispersion and catalytic surface chemistry. The optimized catalyst (0.5% Pt‐3% Sn‐2% Eu on γ‐Al_2_O_3_) achieves a 40.6% propylene yield at 575 °C and a low deactivation rate (0.047 h^−1^), under conditions relevant to industrial practice. Our findings offer a new strategy for designing high‐performance diluted alloy catalysts through rare‐earth promotion, applicable to other dehydrogenation and hydrocarbon upgrading reactions where coke suppression and acid–base balance are critical.

## Graphical abstract

Eu‐promoted PtSn_2_ catalysts leverage the redox activity of Eu and tailored acidity to reduce coke formation, enhancing selectivity and stability in propane dehydrogenation. This synergy significantly improves propylene yield and extends catalyst lifespan, providing an effective strategy for efficient PDH processes.

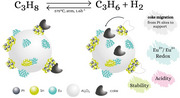

## Introduction

Propylene is a vital component for numerous industrial applications, such as polymer and petrochemical production.^[^ ^1^, ^2^ ^]^ The global propylene market is expected to grow significantly in the coming years, projecting a 165 million tons by 2030.^[^ ^3^, ^4^ ^]^ As petrochemical feedstocks gradually move away from naphtha and C3 volumes decline, the need for alternative starting materials and more energy‐efficient processes for propylene production is escalating. This underscores the global relevance of our research in developing catalysts for nonoxidative propane dehydrogenation.

Propylene, the second most produced chemical in the petrochemical industry,^[^ ^5^ ^]^ is traditionally obtained from oil cracking, which yields a propane–propylene mixture.^[^ ^6^ ^]^ However, C3 separation is a complex, energy‐intensive process with a significant carbon footprint.^[^ ^2^ ^]^ In contrast, nonoxidative propane dehydrogenation (PDH), a direct conversion process from propane to propylene, offers a promising route to pure and economically competitive propylene, surpassing conventional methods.^[^ ^7^, ^8^ ^]^ Next‐generation PDH processing is expected to enhance carbon efficiency significantly due to improved propylene selectivity and reduced coke formation, lowering energy costs in downstream propylene purification.^[^ ^9^ ^]^

**Figure 1:**
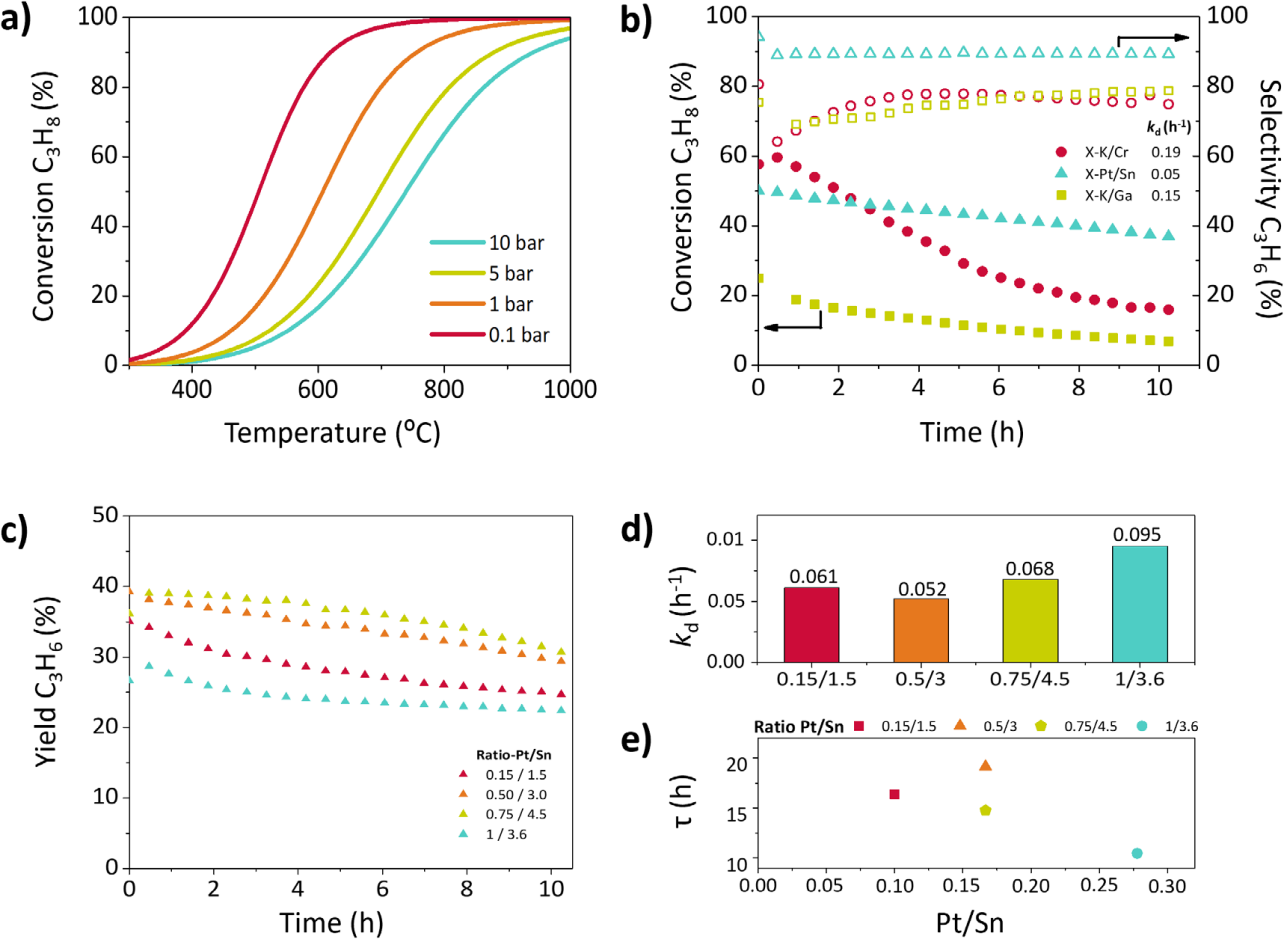
a) Pressure and temperature dependence of C_3_H_8_ equilibrium conversion in the PDH reaction (side reactions not considered); b) C_3_H_8_ conversion (filled symbols, left) and C_3_H_6_ coke‐free selectivity (empty symbols, right) during PDH at 575 °C, WHSV = 1.6 h^−1^, and C_3_H_8_:N_2 _= 12:3 mL·min^−1^ and deactivation coefficients c) C_3_H_6_ yield at same operating conditions as (b); d) Deactivation coefficient and e) Catalyst life, all plotted against the Pt/Sn ratio from synthesis feed (% wt). For reference, the actual Pt/Sn ratios measured by ICP‐OES are provided in Table S1.

PDH is an endothermic reaction limited by thermodynamic equilibrium (XC_3_ ∼ 50% at 600 °C), 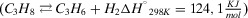.^[^ ^7^ ^]^ The propylene formation is promoted at high operating temperatures (Δ*H_r_* > 0) and low pressures (Δ*n_r_* > 0) as shown in Figure 1a. However, it can lead to severe problems at high temperatures because of coke deposition, sintering of active metal sites,^[^ ^10^ ^]^ and side reactions, including hydrogenolysis, cracking, and isomerization.^[^ ^11^ ^]^ Therefore, much effort has been devoted to alleviating these problems and decreasing the temperature of the process. Different metal‐based catalysts can be found in the literature, Pt, Cr, and Ga being the most reported ones.^[^ ^12^ ^]^ Amongst them, Pt‐based catalysts are widely applied in commercial PDH technology due to their ability to favor C–H cleavage and depict relatively low C–C split yields.^[^ ^12^, ^13^ ^]^ Even though Pt‐based catalysts show high activity in dehydrogenation reactions, there is still room for improving their selectivity and stability. Therefore, the addition of promoters is required to obtain an optimal catalyst. Besides the introduction of Sn and the utilization of alloys containing Pt, significant improvements have also been achieved by adding rare‐earth elements (REs).^[^ ^14^, ^15^ ^]^ Nakaya et al. recently achieved a stable activity for Ca and Pb‐decorated intermetallic PtGa catalyst under H_2_ co‐feeding conditions at 600 °C.^[^ ^16^ ^]^ Considering these results, obtaining a tailor‐made catalyst with minimal deactivation and exhibiting high propylene yield remains challenging.

Herein, we report the influence of adding a third‐metal promoter to the Pt/Sn catalytic system as a strategy to design a competitive and stable catalyst in the PDH reaction without H_2_ co‐feeding. The best results are obtained by an optimized Eu‐promoted Pt/Sn catalyst supported on γ‐alumina, showing propane conversion and propylene selectivity of ∼46% and ∼93%, respectively, at 575 °C, atmospheric pressure, and space velocity (WHSV) of 1.6 h^−1^ at 0.9 h of reaction. Table S1 summarizes the catalytic performance of the reported Pt‐based catalysts for PDH. Considering the amount of Pt loading and the highly concentrated propane feed stream, Eu‐promoted Pt/Sn catalyst shows a superior catalytic performance, which could be attributed to the combined effect of the higher Pt dispersion and the fine‐tuned redox behavior, reached upon introduction of Eu in comparison with other REs promoters.^[^ ^15^ ^]^ Indeed, the particular Eu‐enabled redox and Lewis acidity behavior led to higher catalyst stability and coking suppression, resulting in deactivation constant values of 0.047 h^−1^ after 10 h on stream.

## Results and Discussion

### Benchmarking Cr‐, Ga‐ and Pt‐Based Catalysts for Propane Dehydrogenation

Three reference catalysts based on the most studied classes (Cr, Pt, Ga) were prepared by incipient wet method (see, Table S2) and tested as a first evaluation and optimization study at 575 °C and 1.6 h^−1^. The testing conditions were previously selected from the propylene‐yield optimization study carried out with the K/Cr catalyst (Figure S1) and were subsequently validated in the broader operating‐condition screening presented later in Figure 4. γ‐Al_2_O_3_ was selected as a catalyst support since it offers a higher specific area, aside from well‐balanced Lewis acid sites, leading to adequate dispersion of the active metal phase.^[^ ^17^ ^]^ A comparison of their performance in the PDH reaction (Figure 1b) shows that although the Ga‐based catalyst offers slow deactivation, the conversion values (≈20%) are far from equilibrium (≈38.2%). The rate‐limited desorption of hydrogen species might cause the low activity of Ga‐based sites to form gaseous H_2_.^[^ ^18^ ^]^ On the other hand, the Cr‐based catalyst shows very high conversion values in the early stages of the reaction (≈55%), but faster deactivation. This severe deactivation could originate from coke formation and occupation of the active sites by propylene, primarily propane, which is bound more strongly to Cr sites.^[^ ^19^ ^]^ The coke‐free selectivity for both Cr and Ga‐based catalysts is around 82%, with similar characteristics. Pt‐based catalyst stands out since it shows the highest yield (≈40%) and coke‐free selectivity (≈90%) after 10 h on stream and the slowest deactivation over time (*k*_d_ = 0.052 h^−1^). Therefore, the formation of cracking products is less favored over Pt/Sn catalysts. The discrepancies between equilibrium propane conversion (Figure 1a) and experimental conversions (Figure 1b) are mainly attributed to propane cracking reactions and coke formation as no side reactions are considered in the theoretical equilibrium*.

Combining Pt with other metals to form alloys or Pt–M composites efficiently improves PDH activity and selectivity at lower Pt loading. Sn is the most promising promoter, playing two principal roles in the catalytic mechanism. A first geometric effect^[^ ^20^ ^]^ helps to separate larger Pt particles into smaller clusters, i.e., enhancing dispersion, thus minimizing the population of sites with high coking activity. Second, an electronic role simultaneously promotes the desorption of propylene and coke transfer from Pt sites to the support due to the strong electron transfer between the Pt and Sn atoms.^[^ ^21^, ^22^ ^]^ Density Functional Theory (DFT) calculations^[^ ^19^, ^23^ ^]^ revealed that Sn interaction with Pt‐based sites increases the activation energy for deep propylene dehydrogenation reactions and lowers the desorption barrier of propylene to the gas phase, resulting in an enhanced selectivity toward propylene.^[^ ^24^, ^25^ ^]^

### Tuning the Pt/Sn Ratio to Optimize Catalytic Performance

The Pt/Sn ratio (%wt.) has a pivotal influence on the catalytic PDH performance (Figure 1c) and deactivation rates (Figure 1d,e). The ratio Pt/Sn = 0.5/3 (orange) is identified as an optimal composition reaching the most stable activity. It shows the most extended catalyst life (τ) and the lowest deactivation constant (*k*_d_), in addition to a propylene yield close to the highest value. Increasing (blue) or decreasing (red) Pt/Sn ratio leads to lower propylene yields. It is also remarkable that the strong deactivation experienced by the Pt/Sn = 1/3.6 ratio, indicating that the amount of Sn was not enough to reach a balanced Pt dispersion that mitigates the coke formation, even leading to worse catalytic performance than the Pt/Sn = 0.15/1.5 ratio, which contains half the amount of Pt. Finally, the Pt/Sn = 0.75/4.5 ratio shows the highest propylene yields. Still, it is equilibrium‐limited, and the higher Pt loading also promotes side reactions and coke formation, which are not equilibrium‐limited, resulting in a larger deactivation constant and lower selectivity (see Figure S2) than Pt/Sn = 0.5/3. Therefore, 0.5% Pt and 3% Sn by weight were selected as the optimal composition to further improve the catalyst stability and propylene yield by adding a third promoter.

### Impact of Rare‐Earth Elements on the Activity of Pt–Sn Catalysts

Assuming every surface Pt atom is an active site, its reaction rate is directly proportional to the metal dispersion.^[^ ^26^ ^]^ Here, enhancing metal dispersion,^[^ ^27^, ^28^, ^29^ ^]^ i.e., the surface concentration of active sites, and inhibiting coke formation emerge as primary objectives when introducing an additional promoter to Pt/Sn catalysts. REs were selected as promoters in the studied system with two goals: to boost metal dispersion and fine‐tune the interaction of the catalyst support and Pt/Sn active phase. The selected elements were La, Ce, Eu, Nd, Sm, Gd, and Tb as lanthanides, Y as a transition metal, and In, which shares several characteristics despite not belonging to this group. Previous studies revealed an essential influence of adding different REs on the formation and stability of the Pt‐Sn alloy, primarily due to the change of the metal‐support interaction.^[^ ^30^, ^31^ ^]^ Sequential incipient wetness and co‐impregnation methods were used to prepare the samples (see Supporting Information for experimental details and composition in Table S3). In all cases, the XRD diffraction patterns show the γ‐Al_2_O_3_‐characteristic peaks (Figures S3 and S4). The metallic phase of Pt or its alloys with Sn is undetectable as its characteristic peaks overlap with the prominent peaks of alumina at 39.5° and 45.9°. Additionally, the peaks corresponding to Sn and REs, or their alloys, are also undetectable due to both their low concentration and significant dispersion on the support.

**Figure 2:**
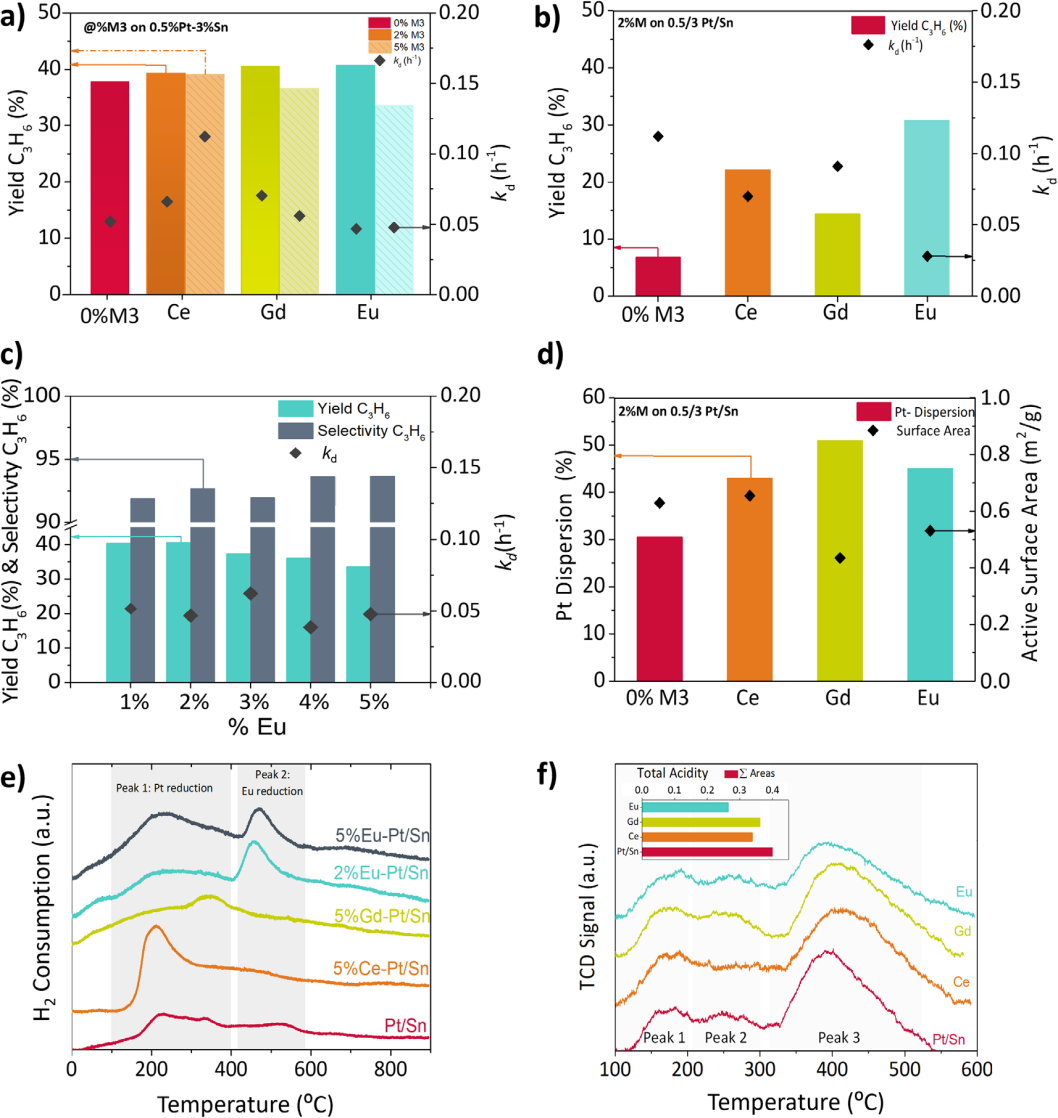
a) PDH deactivation rate constant and yields (at 0.9 h on stream, 575 °C, WHSV = 1.6 h^−1^ and C_3_H_8_:N_2 _= 12:3 mL·min^−1^) shown by 5 wt. % metal loading over 0.5%Pt/3%Sn γ‐Al_2_O_3_ catalyst. Note: Pt/Sn and γ‐Al_2_O_3_ are omitted, and only the % of the additional element is expressed; b) deactivation rate constant and yields at different space velocities, WHSV = 8.0 h^−1^ shown by 2 wt. % metal loading catalysts in PDH reaction; c) %Eu added against the obtained deactivation and yields and selectivities results yields (same operating conditions as a); d) CO chemisorption: Pt dispersion (bars) and active surface area (triangle); e) H_2_‐TPR profiles of some catalyst samples. The corresponding experimental H_2_ volume consumption and peak temperatures are listed in Table S4; f) TPD‐NH_3_ analysis corresponding deconvolution and NH_3_ consumption per peak are summarized in Figure S9, Table S5.

Figure 2a summarizes and compares the catalytic performance at 575 °C and 1.6 h^−1^ after adding Ce, Gd, and Eu (2 or 5%) over a γ‐Al_2_O_3_‐supported 0.5%Pt/3%Sn. The selected promoters and the different loadings cause distinct effects on catalyst performance (Figures 2a and S5). When 5% is added, Ce enables the improvement of the propylene yield. In contrast, Eu and In‐promoted catalysts are the most stable under these conditions, reaching the lowest deactivation rate constants (*k_d_*), i.e., 0.048 and 0.040 h^−1^, respectively, with lower propylene yield than the unpromoted 0.5%Pt/3%Sn. Together with the promoter nature, the loading is crucial to optimize the catalyst performance, as reported in previous works.^[^ ^12^, ^32^ ^]^ In the case of Ce and Gd promoters, reducing the loading to 2% leads to both higher initial propylene yields and deactivation rates. Results for Tb, Y, La, In, Nd, and Sm promoters are shown in Figure S5. A different trend is observed for Y; a lower loading worsens propylene yield and stability. Although Nd shows a higher initial yield (∼41%), the stability slightly worsens, and Sm does not show better results than the already tested elements. The best results are exhibited by 2% Eu, where yield increases by 21% compared to the 5% Eu catalyst, and the deactivation rate remains almost unaltered.

It is noteworthy that, given the optimal operating conditions, the impact of the promoter might not be readily discernible due to constraints imposed by chemical equilibrium. Consequently, a deliberate departure from these optimal conditions is undertaken to elucidate the promoter effect on catalyst performance better. Figure 2b shows the effect of the promoter away from equilibrium—with a much higher WHSV (8 h^−1^), unveiling the superior promotion effect of Eu over the rest of the promoters, exhibiting the highest propylene yield and stability. The detailed study of the Eu loading (Figure 2c, propylene yield, selectivity, and *k*_d_ at 0.9 h on stream) reveals the highest stability (*k*_d_ ∼0.039 h^−1^) for 4% Eu, whereas the maximum yield is observed for the 2% and 1% Eu loadings.

The improved activity and stability achieved by promoting the Pt/Sn system are associated with higher Pt dispersion, tailored redox activity, and surface chemistry. CO chemisorption measured Pt dispersion (Figure 2d), showing an increase with each promoter. In contrast, the catalyst surface area (N_2_‐sorption) barely changes (Figure 2d). The increase in dispersion alone cannot explain the enhanced performance of some catalysts as it does not follow a direct relationship. The addition of REs influences catalyst acidity and interfacial interactions between the metal and the support, playing a crucial role in the improvements obtained. ^[^ ^15^, ^33^ ^]^ Temperature‐programmed reduction analysis (TPR) enables us to characterize the catalyst redox behavior (Figure 2e and Table S4) and reveals the interactions between the metal and the support. A broad reduction peak can be identified within the 100–400 °C range for all catalysts, which can be attributed to the concurrent reduction of Pt and Sn oxidized species.^[^ ^32^, ^34^ ^]^ This reduction is shifted toward lower temperatures for Ce and Eu, indicative of a higher Pt dispersion, higher surface oxygen reducibility,^[^ ^35^, ^36^ ^]^ and promotion of the Pt species reduction.^[^ ^37^ ^]^ Ce and Eu oxides present more abundant surface lattice oxygen species and enhanced vacancy formation and mobility.^[^ ^38^ ^]^ The Ce‐doped sample presents a sharp reduction peak at 220 °C, which is also related to the reduction of Ce^4+^ to Ce^3+^. Moreover, Eu‐doped samples display a second narrower peak centered at 455–470 °C, associated with the reduction of Eu^3+^ to Eu^2+^. Finally, the Gd‐doped sample hardly shows any improvement over the reducibility of the reference Pt/Sn catalyst. XPS analysis of the reduced and spent catalysts is consistent with the TPR results, indicating that all promoters exhibit the same oxidation state (+3), except for Eu and Ce, which show oxidation states of +2 and +4, respectively (Figures S6 and S7).^[^ ^39^ ^]^ The ratio of Eu^3+^/Eu^2+^ in the reduced catalyst was found to be 1.97 (66.4% Eu^3+^ and 33.6% Eu^2+^), which is very similar to the ratio observed for the spent catalyst, a sign of the stability of Eu^2+^ species in the catalyst. For the Ce‐based catalyst, Ce was detected as a mixture of Ce^4+^/Ce^3+^ on the surface. The 2%Ce‐PtSn as‐prepared catalyst exhibits approximately 42.6% Ce^3+^, while the spent catalyst exhibits an increase in this fraction to ∼56%. The chemistry of Pt and Sn was also assessed from the XPS analysis (Figure S8). Pt was detected solely in its metallic form (Pt^0^), while Sn appeared in two forms: metallic Sn (Sn^0^), likely alloyed with Pt, and fully oxidized Sn^4+^ (SnO_2_). The Pt^0^/Sn^0^ ratio was quantified as 1:1.5.

Lanthanide incorporation markedly alters the acid properties of the catalyst support, as evidenced by NH_3_‐TPD profiles (Figure 2f). All samples display three desorption peaks between 150–400 °C, corresponding to weak, medium, and strong acid sites. Deconvolution of these profiles (Figure S9 and Table S5) shows that Eu addition reduces the intensity and temperature of the peak associated with strong acid sites, indicating their partial neutralization. This reduction in acidity is likely to suppress side reactions and coking by promoting propylene desorption.^[^ ^15^, ^32^ ^]^ FTIR analysis of pyridine‐adsorbed samples (Figure S10 and Table S6) further confirms a decrease in Brønsted acidity and an increased Lewis/Brønsted (L/B) ratio across all desorption temperatures (200–400 °C)†. A significant drop in retained pyridine (µm g^−1^) at elevated temperatures also points to weaker acid strength.^[^ ^37^, ^38^ ^]^ These results suggest that Eu mitigates acid‐catalyzed deactivation by suppressing Brønsted acid sites responsible for cracking and arylation, while enhancing Lewis acidity that facilitates olefin desorption.^[^ ^40^, ^41^ ^]^

**Figure 3:**
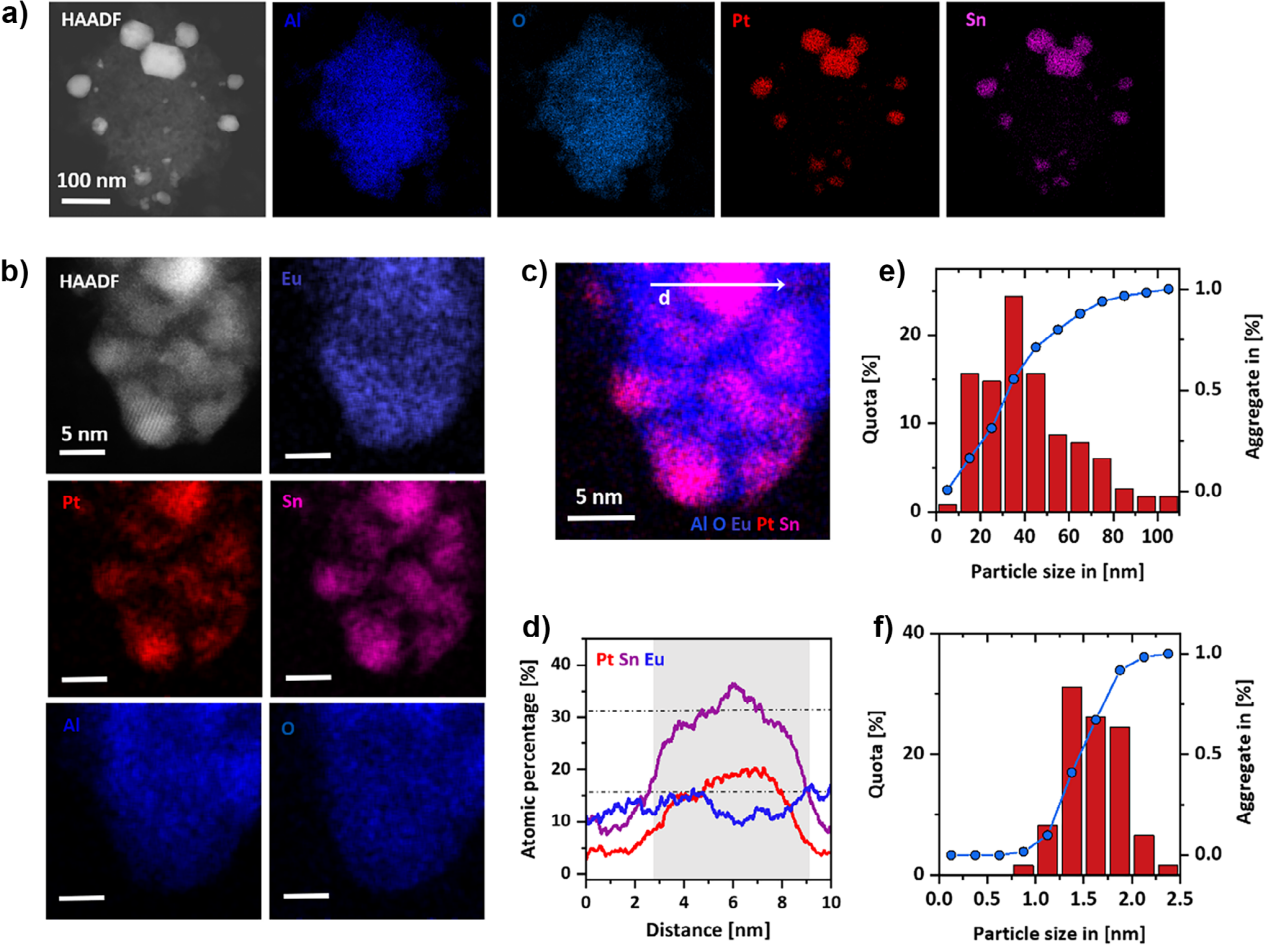
a) STEM‐imaging and corresponding EDS mappings of the reference Pt/Sn catalyst on an γ‐Al_2_O_3_ support. b) STEM‐imaging and corresponding EDS mappings of the 2% Eu promoted Pt/Sn catalyst (mind the strong differences in scale between (a) and (b)). c) Integrated EDS mapping of the Eu‐promoted catalyst. The white arrow highlights the location of the integrated line profile, which is plotted in d). e) Particle size distribution of the reference catalyst based on statistical image analysis. f) Particle size distribution of the Eu‐promoted catalyst.

STEM‐EDS analysis (Figure 3) of the Pt/Sn catalyst unpromoted (a) and 2% Eu‐promoted (b) enabled the visualization of changes in chemistry and morphology that give rise to the improved performance.^[^ ^42^ ^]^ The active centers in the 2% Eu‐promoted catalyst are found in higher proportions, primarily due to significantly smaller particle sizes than the reference Pt/Sn catalyst, which aligns with the CO chemisorption results. ^[^ ^43^, ^44^ ^]^ The analysis of particle size distribution in both catalysts (Figures 3e,f and S17) reveals that the Eu‐promoted catalyst presents astonishingly smaller particle sizes (∼1.3 nm) compared to the unpromoted (35 nm). The EDS mappings in Figure 3b show that Eu is either included in the γ‐Al_2_O_3_ support or on the support surface, but an increase of Eu in the Pt/Sn alloy particles could not be detected. A detailed investigation of the composition of one catalyst particle through a standard less quantification of the EDS mapping (Figures 3c,d and S18) helps to estimate the composition, making the formation of a PtSn_2_ alloy most probable. The Pt^0^/Sn^0^ ratio (1:1.5) obtained by XPS further supports the hypothesis of PtSn_2_ alloy formation. These structural and compositional modifications correlate well with the catalytic behavior, as evidenced in Figure S19, where the Eu‐promoted catalyst exhibits higher C_3_H_8_ conversion and coke‐free selectivity to C_3_H_6_ compared to the unpromoted sample under identical conditions (575 °C, WHSV = 1.6 h^−1^, C_3_H_8_:N_2_ = 80:20), in agreement with the improved dispersion observed.

Based on the combined STEM‐EDS, XPS, H_2_‐TPR, and acidity analyses, we propose a structural model for the Eu–PtSn_2_/γ‐Al_2_O_3_ catalyst, where Eu is mainly dispersed on the support and at the metal–support interface, stabilizing ultrasmall PtSn_2_ nanoparticles (∼1.3 nm) and tuning the acid–base and redox properties (Eu^3+^/Eu^2+^). This schematic is shown in the Supporting Information (Figure S20 and TOC image).

### Coke Formation on RE‐Doped Pt–Sn Catalysts

In addition to the amount of coke formed, catalyst stability is also influenced by the nature of the carbon deposits and their location, whether on the active metal sites or the support. Thermogravimetric analysis (TGA) in air of spent catalysts (Table S7) confirms that the extent of coke formation correlates with both catalyst acidity and the observed deactivation rates. The deactivation rate constant scales with accumulated coke, expressed as mol C per mol Pt (Figure S11b), and aligns with propylene yield trends (Figure S5). Generally, increased coke loading shortens catalyst lifetime (Figure S11a). Raman spectroscopy provides further insight into the structural and compositional nature of coke deposits. Across all catalysts, two dominant features are observed in the Raman spectra (Figure S12, Table S8): the D‐band (∼1000–1500 cm^−1^), associated with disordered (amorphous) carbon, and the G‐band (∼1500–1700 cm^−1^), indicative of graphitic carbon. ^[^ ^45^, ^46^, ^47^, ^48^ ^]^ The similar I_D_/I_G_ ratios across the catalysts suggest a comparable degree of carbon disorder and graphitization. Consequently, differences in catalyst stability are likely attributable not to the nature of the coke but to its location, i.e., whether deposited on the metal (Pt) or the support. Oxidation analysis of spent catalysts (Figure S13 and Table S9) offers further kinetic insights into coke formation, consistent with thermogravimetric trends and deactivation constants. The oxidation profiles feature a broad peak centered at ∼450 °C, reflecting distinct coke oxidation pathways tied to differences in coke formation kinetics and dynamics.^[^ ^27^ ^]^ Two primary processes can be discerned: one at ∼425 °C, attributed to carbon on metal sites, and another at ∼500 °C, corresponding to carbon deposited on the support. Among the catalysts, 2%Eu‐PtSn exhibits the highest proportion of support‐associated coke relative to total carbon, correlating with lower overall coke formation. This suggests enhanced coke migration to the support, thereby mitigating catalyst deactivation. The high carbon tolerance of the Eu‐modified catalyst is further corroborated by XPS analysis (Figure S14) of the survey spectra and the C 1s core‐level emission spectra, revealing a clear trend in surface carbon accumulation: 2%Eu‐PtSn < 2%Gd‐PtSn < 2%Ce‐PtSn. Notably, the 2%Eu‐PtSn catalyst generates carbon at an exceptionally reduced rate under reaction conditions.

### Optimising Operation and Regeneration

**Figure 4:**
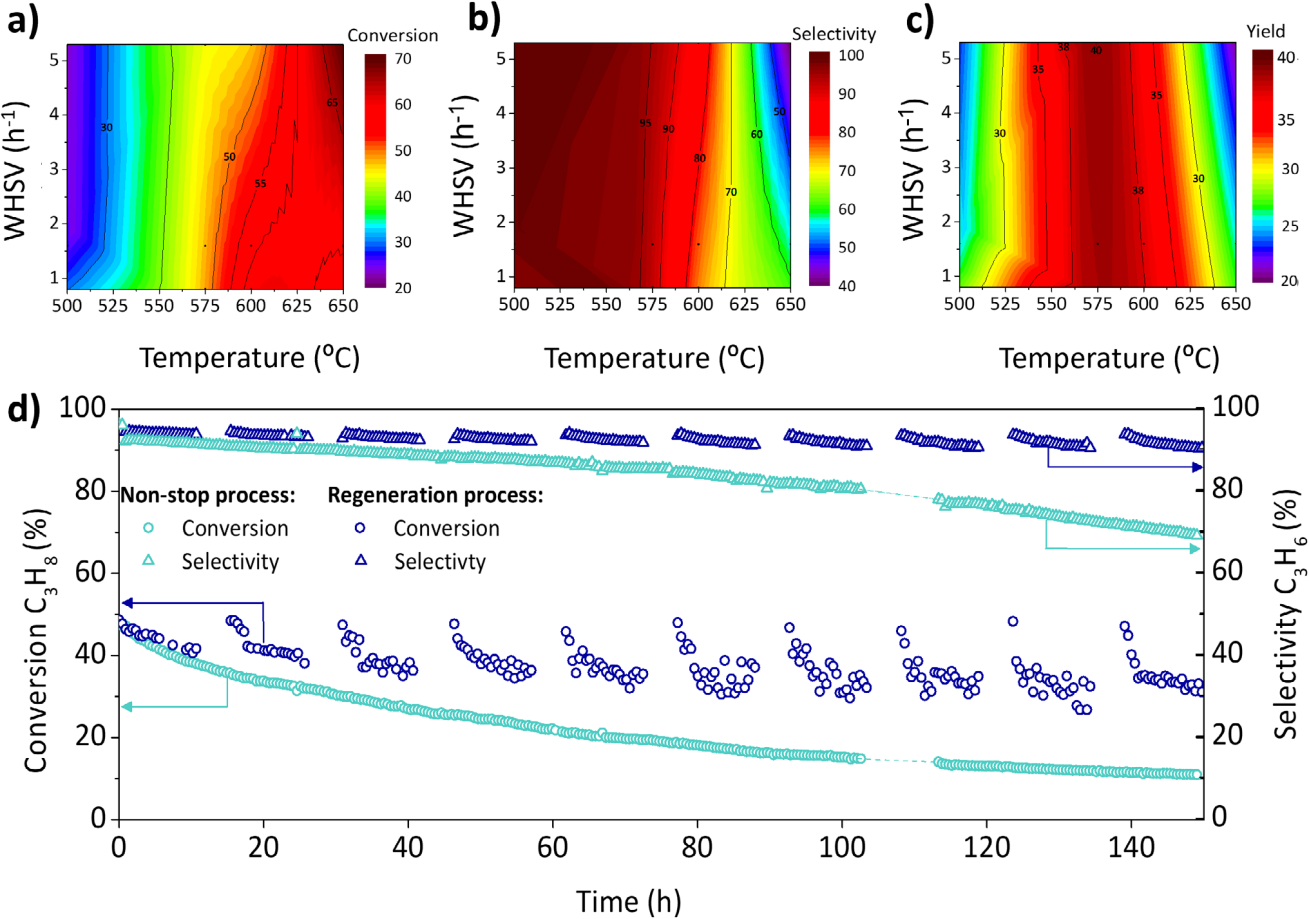
a) Conversion, b) Selectivity, and c) Yield as a function of temperature and WHSV (h^−1^) at initial reaction time (0.9 h) for 0.5%Pt‐3%Sn‐2%Eu γ‐Al_2_O_3_ catalyst; d) Long‐term PDH reaction for 150 h using a 0.5%Pt‐3%Sn‐2%Eu γ‐Al_2_O_3_ catalyst at 575 °C, WHSV = 1.6 h^−1^, and a C_3_H_8_:N_2_ ratio of 12:3 mL·min^−1^, shown in light blue, versus regeneration cycles, dark blue. Each cycle includes 10.6 h of reaction (under the same conditions), 4 h of regeneration with air at 50 mL min^−1^, and 2 h of reactivation with H_2_: Ar at a 1:1 ratio.

The superior performance of the Eu‐modified catalyst is attributed to its higher Pt dispersion, distinctive redox behavior, and reduced acidity, which together suppress coking and enhance propylene yields, setting it apart from catalysts modified with other metals. To further optimize catalytic performance, we investigated the reaction conditions using the most promising formulation, 0.5%Pt‐3%Sn‐2%Eu γ‐Al_2_O_3_. Temperature sweeps were performed between 500 and 650 °C, and WHSV was evaluated from 0.1 to 5 h^−1^. According to thermodynamics, higher temperatures and increased space velocity lead to higher conversion rates (Figure 4a), whereas lower temperatures and moderate WHSV enhance selectivity (Figure 4b). The optimal propylene yield, reflecting a compromise between these parameters, was achieved at 575 °C and 1.6 h^−1^ WHSV (Figure 4c), corresponding to a conversion of 46%, selectivity of 93%, and a propylene yield of 41%.

An extended catalytic study was performed using the optimal operating conditions and pure propane as feed (Figure 4d). The catalyst shows a high propylene yield with moderate deactivation over time, outperforming comparable long‐term studies reported in the literature (Table S1). Figure 4d contrasts a continuous process—operated without regeneration—with a cyclic regeneration strategy applied over 150 h. In the absence of regeneration, propane conversion declined steadily from ∼37% to ∼15% over 145 h on stream, while selectivity remained relatively stable, decreasing slightly from ∼85% to ∼80%. In contrast, the regeneration process—comprising 10.6 h of reaction, followed by 4 h of oxidative regeneration (air) and 2 h of reactivation (H_2_)‐ effectively mitigated deactivation, maintaining higher conversion levels (∼50% to ∼30%) and improved selectivity (∼90% decreasing to ∼85%). It is important to highlight that, in each cycle, the initial values of conversion return to their starting points. Therefore, the deactivation could mostly be ascribed to coking, while globally, some sintering mechanisms may have also contributed to catalyst deactivation. This approach resulted in a ∼45% reduction in the overall deactivation constant (Figure S15). Notably, no co‐feeding of H_2_ or H_2_O was employed to suppress coke formation. Separate experiments with H_2_ co‐feeding showed no improvement in catalyst stability (Figure S16), indicating that the regeneration process is the best option for maintaining reaction yields at industrially productive levels.^[^ ^49^, ^50^ ^]^

## Conclusions

In summary, we report a novel PtSn_2_‐based catalyst for the propane dehydrogenation (PDH), exhibiting exceptional catalytic activity and stability. The incorporation of REs promoters into the PtSn alloy notably enhances performance by increasing alloy dispersion, modulating Lewis acidity, leveraging redox properties, and facilitating coke migration from the active sites to the support. Among the rare‐earth elements tested, Eu is particularly effective. Beyond performance metrics, it is worth noting that Eu exhibits a unique combination of structural and electronic properties among the rare‐earth elements tested. Unlike Ce, Gd, Sm, Nd, Tb, Y, or In, Eu stands out for its stable Eu^2+^ state, half‐filled 4f shell, anomalously high third ionization energy, and strong basicity of Eu^2+^ compounds, making it chemically closer to alkaline earths in its +2 state. This dual Eu^3+^/Eu^2+^ redox flexibility, as confirmed by XPS and H_2_‐TPR, introduces a distinctive electronic modulation of Pt via the Eu^+3^/Eu^+2^ redox couple, enhancing metal–support interactions, promoting Pt and Sn reduction. As a result, Eu uniquely stabilizes ultrasmall PtSn_2_ nanoparticles (∼1.3 nm), fine‐tuning the catalyst acidity—decreases Brønsted acidity while increasing the Lewis/Brønsted ratio—and strongly suppresses coke formation by shifting deposition from Pt sites to the support. These properties culminate in superior dehydrogenation activity and suppress coking. A 2 wt% Eu loading yields the optimal catalyst composition (0.5%Pt‐3%Sn‐2%Eu over γ‐Al_2_O_3_), achieving a propylene yield of 40.6% after 0.9 h on stream at 575 °C and 1.6 h^−1^, with a deactivation rate of 0.047 h^−1^. Future efforts will focus on regeneration strategies to assess industrial applicability. These findings unveil a new approach for designing robust diluted noble‐metal alloy catalysts, such as PtSn_2_, by synergistically engineering acid–base and redox characteristics while maximizing metal dispersion.

## Conflict of Interests

The authors declare no conflict of interest.

## Supporting information



Supplementary Information

## Data Availability

The data that support the findings of this study are available from the corresponding author upon reasonable request.

## Footnotes

* Equilibrium data based on the UNIFAC property method with the Redlich‐Kwong equation of state and Henry's law

† Adsorption peaks around 1450 cm^−1^ and 1600 cm^−1^ are associated with Lewis acid sites,^[^ ^51^ ^]^ while the peak at 1541 cm^−1^ are related to Brønsted acid sites.^[^ ^51^ ^]^ The peak at 1490 cm^−1^ was attributed to the interaction with both acid sites
