# Supplementary material for: Redox‐Acidity Interplay in Eu‐Promoted PtSn2 Catalysts for Selective and Stable Propane Dehydrogenation
Source: Angew Chem Int Ed Engl. 2025 Oct 23;64(50):e202512853. doi: 10.1002/anie.202512853 (PMC12684326; doi:10.1002/anie.202512853)
Supplement: Supplementary file 1 — Supplementary Information [file ANIE-64-e202512853-s001.pdf]

Redox-Acidity Interplay in Eu-promoted $PtSn_2$ Catalysts for Selective and Stable Propane Dehydrogenation

María I. Valls[a], Jesús Ara[a], Sonia Escolástico[a], Sonia Remiro-Buenamañana[a], David Catalán-Martínez[a], Julien Grand[b], Moritz Kindelmann[c], Joachim Mayer[c,d], Simona Somacescu[e], Daniel Curulla-Ferré[b], and Jose M. Serra*[a]

[a] Instituto de Tecnología Química, Consejo Superior de Investigaciones Científicas, Universitat Politècnica de València
Valencia, 46022, Spain
E-mail: jsalfaro@upvnet.upv.es

[b] TotalEnergies S.E, Zone Industrielle Feluy C, B7181 Seneffe, Belgium

[c] Ernst Ruska-Center for Microscopy and Spectroscopy with Electrons (ER-C), Forschungszentrum Jülich GmbH, 52425 Juelich, Germany

[d] Central Facility for Electron Microscopy (GFE), RWTH Aachen University, 52074 Aachen, Germany

[e] c"Ilie Murgulescu" Institute of Physical Chemistry, Romanian Academy, Spl. Independentei 202, 060021 Bucharest, Romania

Supporting information for this article is given via a link at the end of the document.

Materials and methods

Catalyst preparation - Preparation of catalyst supported on ϒ-Al_2O_3

The catalysts employed in this investigation are prepared using the sequential co-impregnation incipient wetness method [1]. Gamma Al_2O_3 serves as the joint support. Sn and the promoter metals (M3) are initially co-impregnated, diluted in corresponding ethanol, and dried at 120 °C for 2 h. Subsequently, the resultant material is gently ground before the second metal impregnation, involving Pt, with water used as a solvent, followed by another drying process. Ultimately, the calcination step occurs in a furnace at atmospheric pressure, reaching 550 °C for 5 h with a heating rate of 2 °C/min. For reference, all the chemicals utilized are outlined in Table S2.For the catalysts of K/Cr and K/Ga, both sequential impregnations use water as a solvent, first K and later Cr or Ga.

The actual Pt, Sn, and M_3 loadings of the catalysts were quantified by inductively coupled plasma–optical emission spectroscopy (ICP-OES) and are reported in Table S1. For simplicity, the Pt/Sn ratios reported throughout the manuscript, including Figures 1c-e, correspond to the synthesis feed ratio in weight percent (% wt.).

Experimental setup – Catalytic testing

The reactions were carried out in a fixed-bed chemical reactor widely used in industrial processes for catalytic reactions. It consists of a fixed bed of catalyst particles through which reactive gases flow. The reactants are converted into products as they flow through the catalyst bed. This design allows efficient contact between the reactants and the catalyst, promoting high conversion rates and selectivity. Catalytic performance was evaluated in a continuous-flow fixed-bed quartz reactor (15 mm inner diameter) housed in a temperature-controlled tubular furnace. The isothermal zone (~4 cm) was filled with 1.25 g of catalyst (sieved to 200–400 µm) diluted with silicon carbide (SiC) in a 1.25:1.75 mass ratio (catalyst: SiC). Prior to each experiment, the catalytic bed (catalyst diluted with SIC) was preheated under Ar flow to 200–250 °C. Catalyst activation was carried out in situ under H_2 flow by ramping the temperature to 575 °C and holding for 2 h.

A reference reaction was performed at atmospheric pressure using a total gas flow of 15 $mL \cdot min^{-1}$ and a feed composition of 80:20 vol.% C_3H_8:N_2, with N_2 serving as an internal standard for volume expansion during reaction. The weight hourly space velocity (WHSV) was maintained at 1.6 h^{-1} at 575°C.

The reactor effluent was analyzed online using a gas chromatograph (Bruker GC-450) equipped with two thermal conductivity detectors (TCD) using Haysep-Q, Haysep-D, and Molsieve 13X columns, and one flame ionization detector (FID) with an HP-Plot Al_2O_3 SS column for C_3 hydrocarbon separation. Details of the calculation of the performance parameters, such as propane conversion and propylene selectivity, are provided in the Supplementary Methods.

Physicochemical characterization of catalysts

X-ray diffraction (XRD)

X-ray diffraction (XRD) is a non-destructive technique that identifies crystalline phases and crystalline materials' orientation, lattice parameters, crystallite size, and thermal expansion. X-ray diffraction was used to determine and confirm the crystalline structure of a solid sample. Powder X-ray diffraction (XRD) patterns were collected in a PANalytical CUBIX diffractometer equipped with a graphite monochromator, operating at 40 kV and 45 mA and employing nickel-filtered Cu Kα radiation (λ = 0.1542 nm). XRD patterns were recorded in the 2θ range from 10º to 40º and analyzed using X'Pert Highscore Plus software.

Elemental analysis was performed by ICP-OES (Varian 715-ES) after digestion of 30 mg of catalyst in HNO_3:HF:HCl (1:1:3, v/v/v). *Scanning transmission electron microscopy* (STEM) and energy-dispersive X-ray spectroscopy (EDXS) were carried out using a Hitachi HF5000 and a Thermo Fisher Spectra 300, both equipped with advanced EDS systems. Atomic-scale elemental maps were quantified using standardless protocols (±5% error).

Photoelectron spectra (XPS)

Photoelectron spectra (XPS) were recorded on a SPECS spectrometer by using Al Kα radiation (Al K_α=1486.6 eV), an analyzer pass energy of 30 V, an X-ray power of 100 W and under an operating pressure of 10_{-9} mbar. The binding energy (BE) scale was regulated by setting the C 1s transition at 284.6 eV. The accuracy of the BE was ±0.1 eV. Spectra analysis has been performed using the CASA software.

Thermogravimetric analyses (TGA)

The chemical and thermal stability of materials can be studied using the thermogravimetric (TG) technique, commonly used to investigate decomposition, dehydration, etc. The TG device registers the mass changes undergone by the sample as a function of temperature, time, and atmosphere. Thermogravimetric (TGA) analyses were done on a TGA/SDTA851e (Mettler Toledo) instrument coupled to a thermobalance. The temperature was increased from room temperature up to 800 °C in air at a heating rate of 10 °C·min^{-1}. This study used the TG technique to quantify the coke amount over the catalysts used in the PDH reaction.

Inductively Coupled Plasma Optical Emission Spectroscopy (ICP-OES)

ICP-OES on a Varian 715-ES confirmed and determined the metal content on the catalysts. The solid samples in powder (30 mg) were dispersed in a volumetric mixture of HNO_3:HF: HCl, 1:1:3 (vol. ratio). In all cases, the calibration curve was adjusted to the expected analyte concentration and was determined using standard solutions (Aldrich).

Temperature-programmed oxidation (TPO)

Temperature-programmed oxidation of the catalysts used in the PDH reaction allows the determination of the nature and location of carbonaceous species (coke) therein.

The TPO experiments were carried out on an AutoChem 2920 apparatus from Micromeritics. For TPO analysis, the spent catalyst was introduced, increasing up to 900 °C at a heating rate of 10 °C·min-1 with 50mL/min of gas mixture comprising O_2 and He in a volumetric ratio of 0.5:95 Combustion profiles were monitored using the detector signals.

Temperature-Programmed Reduction (TPR)

Temperature-Programmed Reduction (TPR) is a technique employed to examine the reducibility of metal species, specifically Pt, along with the interactions between the metal and its support material, such as alumina. This study conducted TPR experiments using a Thermo Scientific TPR/TPD system, utilizing a hydrogen/nitrogen (N_2) mixture as the reducing gas.

Nitrogen sorption analysis (Area BET)

N_2 adsorption isotherms were determined at -196 °C on Micromeritics ASAP 2420 equipment. The specific surface areas were obtained using the BET model, and micropore volume was measured by applying the t-plot approach to the adsorption branch of the isotherms.

CO Chemisorption

250 mg of a sieved catalyst sample (0.2–0.4 mm) are inserted into a quartz reactor of a Quantachrome Autosorb-1C equipment and reduced (150 mL min−1 H2 flow at 450 °C for 2 h, 10 °C/min), degassed at 30 °C and under vacuum (1.333·10−3 Pa) for 2 h, and exposed to a CO flow up to saturation to obtain the CO sorption isotherms.

Raman analysis.

In this study, Raman spectroscopy was employed to determine the structural state of coke and, in conjunction with other techniques, to quantify its composition. The model used was a Renishaw Raman spectrometer (New Mills, UK), equipped with high-resolution optics and laser systems, providing detailed molecular insights critical for this analysis.

Scanning transmission electron microscopy (STEM)

Sample preparation for STEM measurements was done by drop casting of ethanol suspended Al_2O_3 supported $PtSn_2$ nanoparticles on a conventional carbon holey grid (Plano GmbH, Germany). STEM imaging and EDXS analysis were done using a probe corrected Hitachi HF5000 microscope (Hitachi High-Tech, Japan), which is equipped with a AztecEnergy EDS System with 2 Ultim Max TLE detectors (Oxford Instruments, UK) and a probe corrected TFS Spectra 300 (Thermo Fischer Scientific, USA) equipped with a Super-X EDS Detector. The processing of the EDS results was done using the Aztec and Velox software package, respectively. The atomic quantification for the high resolution EDS mapping in Fig. 3b,c is done using standardless quantification, meaning that absolute values might have larger errors (+- 5%).

TPD-NH_3

NH_3-TPD (Temperature-Programmed Desorption of Ammonia) was employed to characterize the acidity of the catalyst support. This technique provided detailed insights into the distribution and strength of acid sites, distinguishing weak from strong acid sites based on ammonia desorption temperatures. The analysis was performed using Thermo Scientific TPR/TPD system with precise temperature control and a thermal conductivity detector (TCD), enabling the quantification of acid site density and strength, essential for understanding catalytic performance and optimizing reaction conditions.

Determination of Brønsted and Lewis Acid Sites by Pyridine-Adsorbed IR Spectroscopy

Acidity measurements were conducted using a Thermo Scientific Nicolet iS10 spectrometer in transmission mode. The samples were prepared as 1 cm^2 pellets and underwent a pretreatment at 50 °C prior to pyridine adsorption. The experimental conditions featured a spectral resolution of 4 cm^{-1}, a spacing of 0.482 cm^{-1}, and 32 scans accumulated over a spectral range from 4000 to

400 cm^{-1}. A gain setting of 4 and a scanning speed of 0.6329 cm/s were employed. The distinction between Brønsted and Lewis acid sites was achieved by analyzing the characteristic IR absorption bands corresponding to adsorbed pyridine, with bands around 1540 cm^{-1} assigned to Brønsted sites and those near 1450 cm^{-1} attributed to Lewis sites. The number of acid sites was quantified in micromoles of adsorbed pyridine per gram of catalyst (µmol Py/g), calculated using the molar extinction coefficients reported by Emeis.[2]

Mathematical details of gas products analysis

The reaction products were analyzed with a gas chromatograph (Bruker 450GC). Hydrocarbons were quantified using a flame-ionization detector (FID). Besides propene and hydrogen, cracked products, mainly methane, ethane, and ethylene, are formed. In addition, other side products such as isobutylene, t-2-butene, c-2-butene, or 1-butene are measured, although in a proportion several orders of magnitude less than the cracked products. Each species' molar fraction is quantified based on its peak area recorded in the chromatograph and using N_2 as the internal standard:

$$xN_2 = \frac{Area\ N_2}{FR_{N_2}} \tag{1}$$

$$xH_2 = \frac{Area\ H_2}{FR_{H_2}} \tag{2}$$

$$xC_3H_8 = \frac{Area\ C_3H_8 \cdot xN_2}{Area\ N_2 \cdot FR_{C_3H_8}\ N_2\ based} \tag{3}$$

$$xC_i = \frac{\frac{Area\ C_i}{MW_{C_i} \cdot FID_i} \cdot xC_3H_8}{\frac{FR_{C_3H_8}\ N_2\ based}{MW_{C_3H_8} \cdot FID_{C_3H_8}}} \tag{4}$$

Where C_i includes all the components with carbon atoms in the exit gas stream, $FR_{C_3H_8}$ is the response factor of propane, MW_{C_i} is the molecular weight, and FID_i is a detector factor. Conversion and selectivity of this reactive system are typically calculated as:

$$X_{C_3H_8} = \frac{(F_{C_3H_8})_0 - (F_{C_3H_8})_{out}}{(F_{C_3H_8})_0} \tag{5}$$

$$S_i = \frac{(n_i \cdot F_i)_{out}}{\sum_i (n_i \cdot F_i)_{out}} \tag{6}$$

Where n_i is the number of carbon atoms of component I, and F_i is its molar flow. However, using the abovementioned equations, the flowmeter error might strongly affect the results, especially if the internal standard is insufficient. A mathematical model based on the molar fractions measured in the chromatograph can be developed to overcome this issue. Firstly, the reactive system considered includes the propane dehydrogenation reaction, cracking reactions, and coke formation as follows:

r1	$C_3H_8 \rightarrow C_3H_6 + H_2$
r2	$C_3H_8 \rightarrow C_2H_4 + CH_4$
r3	$C_2H_4 + H_2 \rightarrow C_2H_6$
r4	$C_3H_8 \rightarrow 3C + 4H_2$

Then, some relationships between the species involved can be established:

$$\frac{xC_2H_6}{xCH_4} = \frac{r3}{r2} \tag{7}$$

$$\frac{xCH_4}{xC_3H_6} = \frac{r2}{r1} \tag{8}$$

$$\frac{xC_2H_6}{xC_3H_6} = \frac{r3}{r1} \tag{9}$$

$$\frac{xH_2}{xC_3H_6} = \frac{r1 - r3 + 4r4}{r1} \tag{10}$$

$$\frac{xH_2}{xCH_4} = \frac{r1 - r3 + 4r4}{r2} \tag{11}$$

$$\frac{xH_2}{xC_2H_4} = \frac{r1 - r3 + 4r4}{r3} \tag{12}$$

Substituting Eqs. 7-9 in Eqs. 10-12 and solving:

$$\frac{r4}{r1} = \frac{1}{4}\left(\frac{xH_2}{xC_3H_6} - 1 + \frac{r3}{r1}\right) \tag{13}$$

$$\frac{r4}{r2} = \frac{1}{4}\left(\frac{xH_2}{xCH_4} - \frac{r1}{r2} + \frac{r3}{r2}\right) \tag{14}$$

$$\frac{r4}{r3} = \frac{1}{4}\left(\frac{xH_2}{xC_2H_4} - \frac{r1}{r3} + 1\right) \tag{15}$$

The selectivity towards each reaction can be expressed as:

$$S_{r_i} = \frac{r_i}{\sum_{n \neq i} r_n} = \frac{1}{1 + \sum_{n \neq i}\left(\frac{r_n}{r_i}\right)} \tag{15}$$

$$S_{coke} = 1 - S_{r_1} - S_{r_2} - S_{r_3} \tag{16}$$

And finally, the propane conversion can be calculated as:

$$\frac{xC_3H_6}{xC_3H_8} = \frac{r1}{n0(1-X)} = \frac{n0 \cdot X \cdot S_{r_1}}{n0(1-X)} = \frac{X \cdot S_{r_1}}{(1-X)} \rightarrow \frac{X}{1-X} = \frac{xC_3H_6}{xC_3H_8}\frac{1}{S_{r_1}} \rightarrow X = \frac{\frac{xC_3H_6}{xC_3H_8}\frac{1}{S_{r_1}}}{1+\frac{xC_3H_6}{xC_3H_8}\frac{1}{S_{r_1}}} \tag{17}$$

Table S1. Catalytic performance of reported Pt-based catalyst for PDH reaction.

№	Catalyst	Pt *(wt%)*	T *(°C)*	WHSV[b] *(h^{-1})*	Gas composition (%)	X_0 (%)	X_f (%)	Time on stream (h)	C_3H_6 Selectivity (%)	Reference
1	0.5Pt-3Sn-2Eu-ϒ-Al_2O_3	0.5	575	1.6	C_3H_8/N_2 = 80/20	50.9	38.5	10.5	94.9	This study
2	PtGa-Ca-Pb/SiO_2	3	600	9.8	C_3H_8/He = 33.3/66.6	37.8	24	8	98-96.6	[1]
3	PtGa-Ca-Pb/SiO_2	3	600	5.9	C_3H_8/H_2/He = 33/17/50	37.6	34.4	300	98.3-98.1	[1]
4	Pt/Sn-ZSM-5	0.32	600	1.8	C_3H_8/N_2 = 23/77	70	45	24	90	[2]
5	PtSn$Al_{0.2}$/SBA-15	0.5	590	2.4	C_3H_8/Ar =20/80	55.9	40.5	10	98.5	[3]
6	K-PtSn@MFI-600H2-22h	0.4	600	29.5	C_3H_8/He =24/76	38.7	31.9	25	>97	[4]
7	$Ga^{\delta+}$Pt0 /SiO_2	4.37	550	2.1	C_3H_8/Ar =25/75	40.7	38.5	20	63.5	[5]
8	Pt/Sn- ϒ Al_2O_3	0.5	600	3.2	C_3H_8 = 100	35.6	N/A	6	88.5	[6]
9	Pt/Ga- ϒ Al_2O_3	0.51	575	5.9	C_3H_8/N_2 = 10/90	39	28	3	95	[7]
10	Pt/Sn- ϴ-Al_2O_3	0.5	600	26.5	C_3H_8/H_2 = 60/40	28.8	25.5	5	91	[8]
11	Pt/Sn-Na/1.0La- Al_2O_3	0.5	590	3	C_3H_8/H_2 = 20/80	41.1	37	6	96.2	[9]
12	Pt–Sn/La–Al (Al_2O_3)	3	600	-	C_3H_8/N_2 = 23/77	~ 51	~ 28	3	~ 95	[10]
13	Pt–Sn/Ce–Al (Al_2O_3)	3	600	-	C_3H_8/N_2 = 23/77	~ 40	~ 22	3	~ 94	[10]
14	Pt–Sn/Y–Al (Al_2O_3)	3	600	-	C_3H_8/N_2 = 23/77	~23	~16	3	~ 90	[10]
15	Pt-Sn-Meso Al_2O_3	0.4	590	3	C_3H_8/N_2 = 80/20	30	25	6	92	[11]
16	Pt-Ga/(Ce) Al_2O_3	0.1	620	5.4	C_3H_8/He =20/80	58.5	56.1	0.5	98	[12]
17	CePt/SSF	1	580	4.05	C_3H_8/Ar =20/80	44.9	37.5	33	92.8	[13]
18	InPt/SSF	1	580	4.05	C_3H_8/Ar =20/80	46.9	40.9	33	98	[13]
19	LaPt/SSF	1	580	4.05	C_3H_8/Ar =20/80	44.4	35.2	33	92	[13]
20	FePt/SSF	1	580	4.05	C_3H_8/Ar =20/80	56.8	44	33	89.7	[13]
21	Pt-Sn-Al_2O_3 nanosheet	0.35	590	9.4	C_3H_8/H_2/He = 16/20/64	48.7	44.6	20	98	[14]
22	Pt-Sn-Mg-SBA-15	1	580	8.3	C_3H_8/Ar =70/30	43	38.1	6	97.8	[15]
23	PtLa/mz-deGa	1	580	11	C_3H_8 = 100	42	17	480 (20 days)	95	[16]
24	PtY/mz-deGa	1	580	11	C_3H_8 = 100	42	5	312 (13 days)	94	[16]

The catalyst included here is the best-performing one of the articles considered. [b] WHSV: Weight hourly based on propane gas flow (h^{-1})

Chemicals

Table S2. List of compounds used in the preparation of the catalysts.

Compound	Formula	Company	CAS number
Gamma alumina	γ-Al_2O_3	ABCR	1344-28-1
Tetraammineplatinum (II) chloride hydrate	$Pt(NH_3)_4\ Cl_2\ H_2O$	Merck chemicals	108374-32-9
Tin (II) chloride dihydrate	$SnCl_2\ 2H_2O$	Merck chemicals	10025-69-1
Europium (III) nitrate hexahydrate	$Eu\ (NO_3)_3{\cdot}6H_2O$	Alfa Aesar	10031-53-5
Potassium nitrate	KNO_3	Merck chemicals	7757-79-1
Chromium (III) nitrate nonahydrate	$Cr(NO_3)_3{\cdot}9H_2O$	Merck chemicals	7789-02-8
Gallium (III) nitrate hydrate	$Ga(NO_3)_3{\cdot}H_2O$	Merck chemicals	69365-72-6
Cerium (III) nitrate hexahydrate	$Ce\ (NO_3)_3{\cdot}6H_2O$	Alfa Aesar	10294-41-4
Indium (III) nitrate hydrate	$In(NO_3)_3{\cdot}H_2O$	Alfa Aesar	207398-97-8
Gadolinium (III) nitrate hexahydrate	$Gd\ (NO_3)_3{\cdot}6H_2O$	Alfa Aesar	19598-90-4
Lanthanum (III) chloride hydrate	$LaCl_3{\cdot}H_2O$	Alfa Aesar	20211-76-1
Terbium (III) nitrate hexahydrate	$Tb\ (NO_3)_3{\cdot}6H_2O$	Alfa Aesar	57584-27-7
Samarium (III) nitrate hexahydrate	$Sm\ (NO_3)_3{\cdot}6H_2O$	Merck chemicals	13759-83-6
Yttrium (III) nitrate hexahydrate	$Y(NO_3)_3{\cdot}6H_2O$	Alfa Aesar	13494-98-9

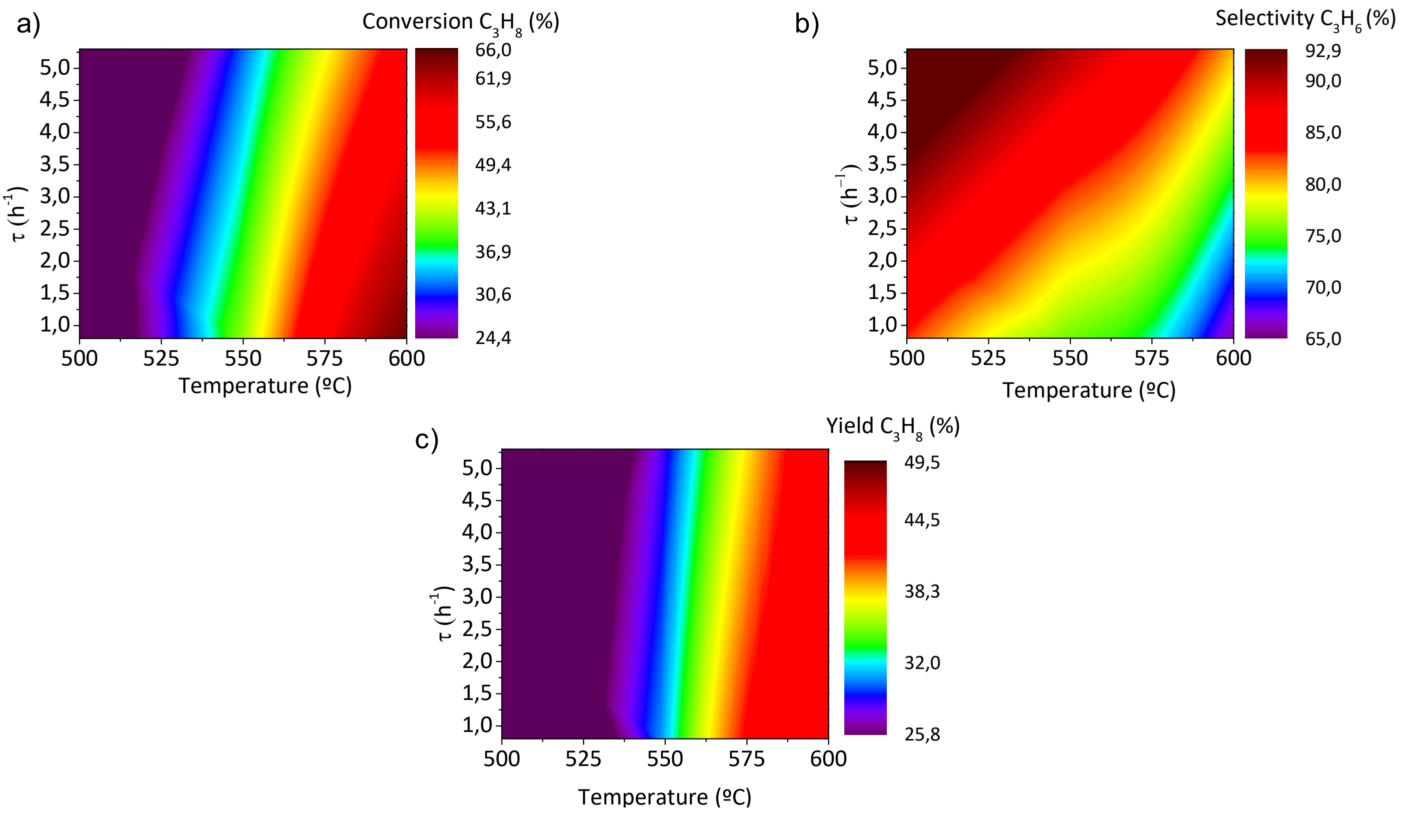

Figure S1: Mapping of operation conditions (temperature and space velocity) in the catalytic PDH. .a) Conversion, b) Selectivity, and c) Yield as a function of temperature and WHSV (h^{-1}) at initial reaction time (0.9 h) for K/Cr- γ-Al_2O_3 catalyst

Identifying the optimal operating conditions for PDH involves screening the temperature and space velocity (WHSV) in separate fixed-bed experiments.

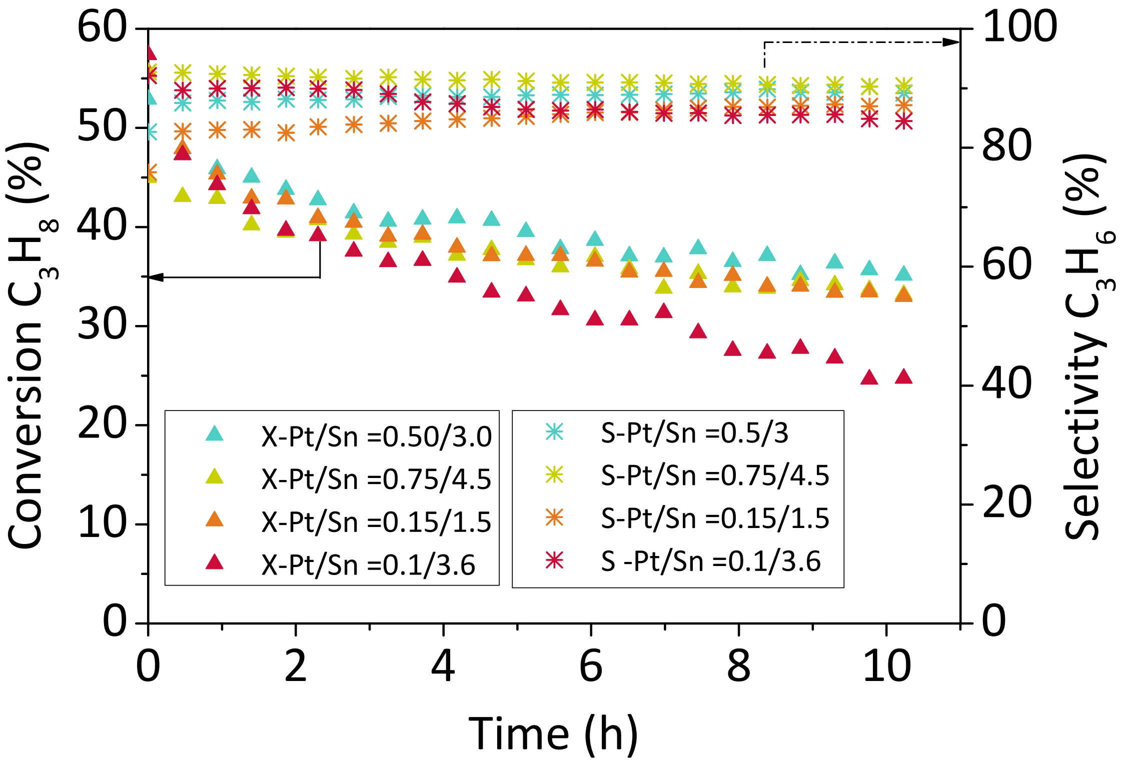

Figure S2. C_3H_8 conversion (solid triangles, left) and C_3H_6 coke-free selectivity (asterisks, right) at 575 °C and WHSV=1.6 h^{-1} for different Pt/Sn ratios. C_3H_8:N_2=12:3mL·min $^{-1}$.

Table S3. ICP analysis results of metal loading in different catalysts.

Sample	Catalyst composition	ICP			
		%Pt	%Sn	%M3	Area (m^2/g)
2% Ce	0.5%Pt/3%Sn/2%Ce-γ-Al_2O_3	0.44	2.62	1.22	194.26
5% Ce	0.5%Pt/3%Sn/5%Ce-γ-Al_2O_3	0.37	2.52	3.17	185.87
1% Eu	0.5%Pt/3%Sn/1%Eu-γ-Al_2O_3	0.40	2.51	0.87	197.89
2% Eu	0.5%Pt/3%Sn/2%Eu-γ-Al_2O_3	0.39	2.49	1.39	198.19
3% Eu	0.5%Pt/3%Sn/3%Eu-γ-Al_2O_3	0.40	2.56	2.43	198.04
4% Eu	0.5%Pt/3%Sn/4%Eu-γ-Al_2O_3	0.38	2.47	3.63	198.59
5% Eu	0.5%Pt/3%Sn/5%Eu-γ-Al_2O_3	0.40	2.51	4.20	193.36
5% Tb	0.5%Pt/3%Sn/5%Tb-γ-Al_2O_3	0.39	2.48	4.33	194.01
5% Y	0.5%Pt/3%Sn/5%Y-γ-Al_2O_3	0.39	2.40	3.09	192.50
5% La	0.5%Pt/3%Sn/5%La-γ-Al_2O_3	0.39	2.49	2.47	198.13
5% In	0.5%Pt/3%Sn/5%In-γ-Al_2O_3	0.39	2.25	3.46	192.07
5% Gd	0.5%Pt/3%Sn/5%Gd-γ-Al_2O_3	0.42	2.45	4.53	192.25
2% Gd	0.5%Pt/3%Sn/2%Gd-γ-Al_2O_3	0.41	2.70	1.49	198.17
2% Nd	0.5%Pt/3%Sn/2%Nd-γ-Al_2O_3	0.41	2.59	0.68	199.82
2% Y	0.5%Pt/3%Sn/2%Y-γ-Al_2O_3	0.43	2.52	1.39	201.10
2% Sm	0.5%Pt/3%Sn/2%Sm-γ-Al_2O_3	0.38	2.61	1.02	203.37

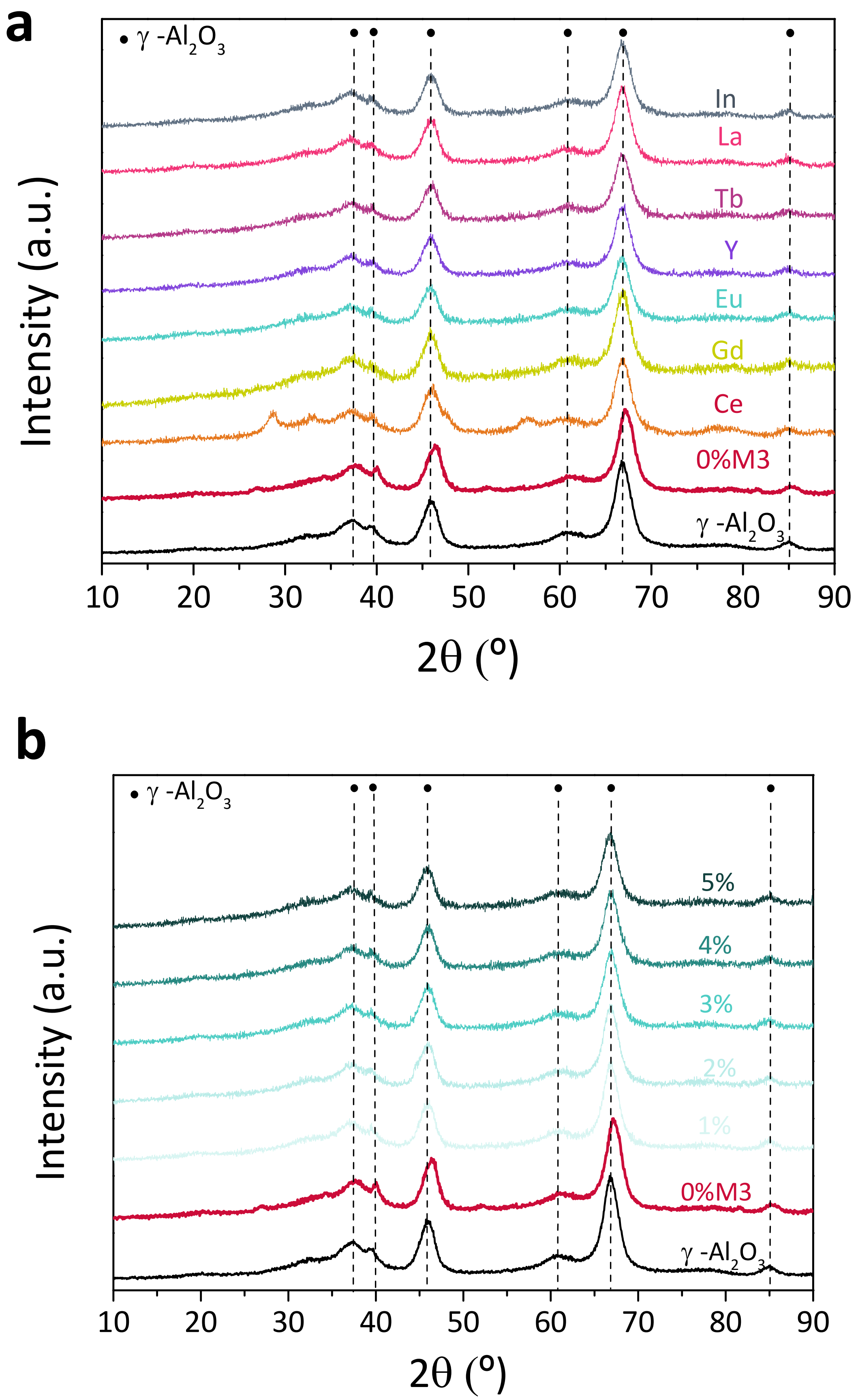

Figure S3. a) XRD diffraction patterns for as-synthesized 5% M3 samples. b) XRD diffraction patterns with as-synthesized samples with different % Eu.

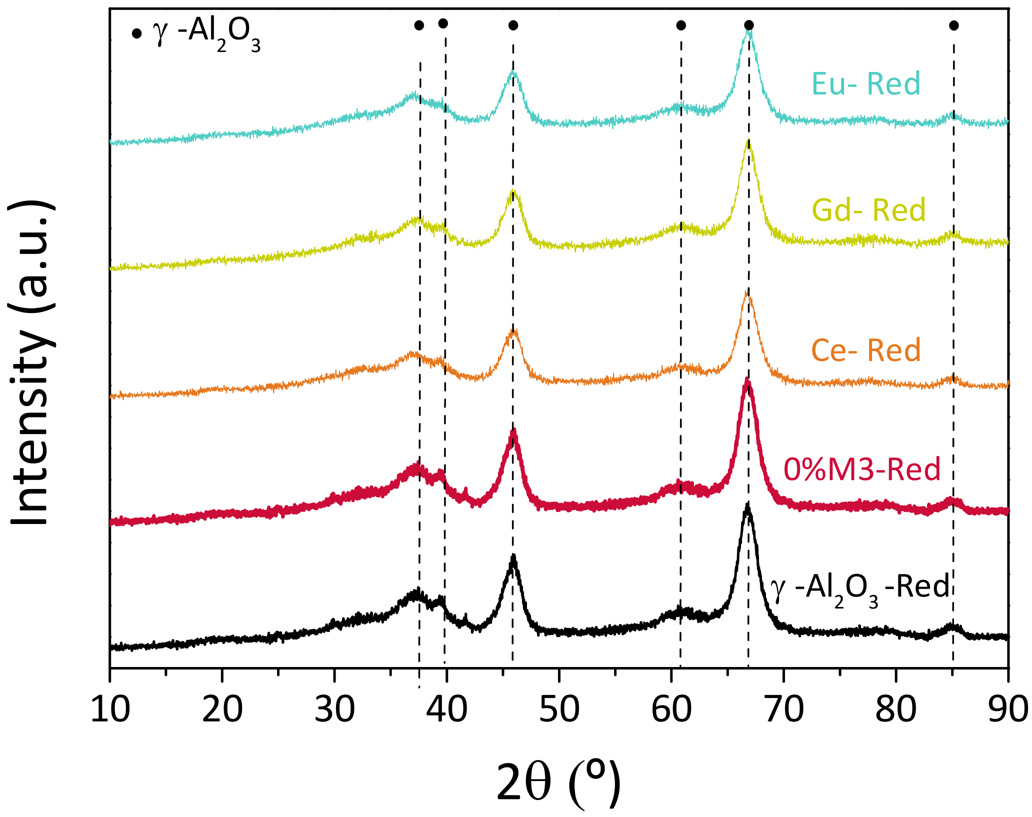

Figure S4. XRD diffraction patterns for reference catalysts reduced for 180 min in H_2.

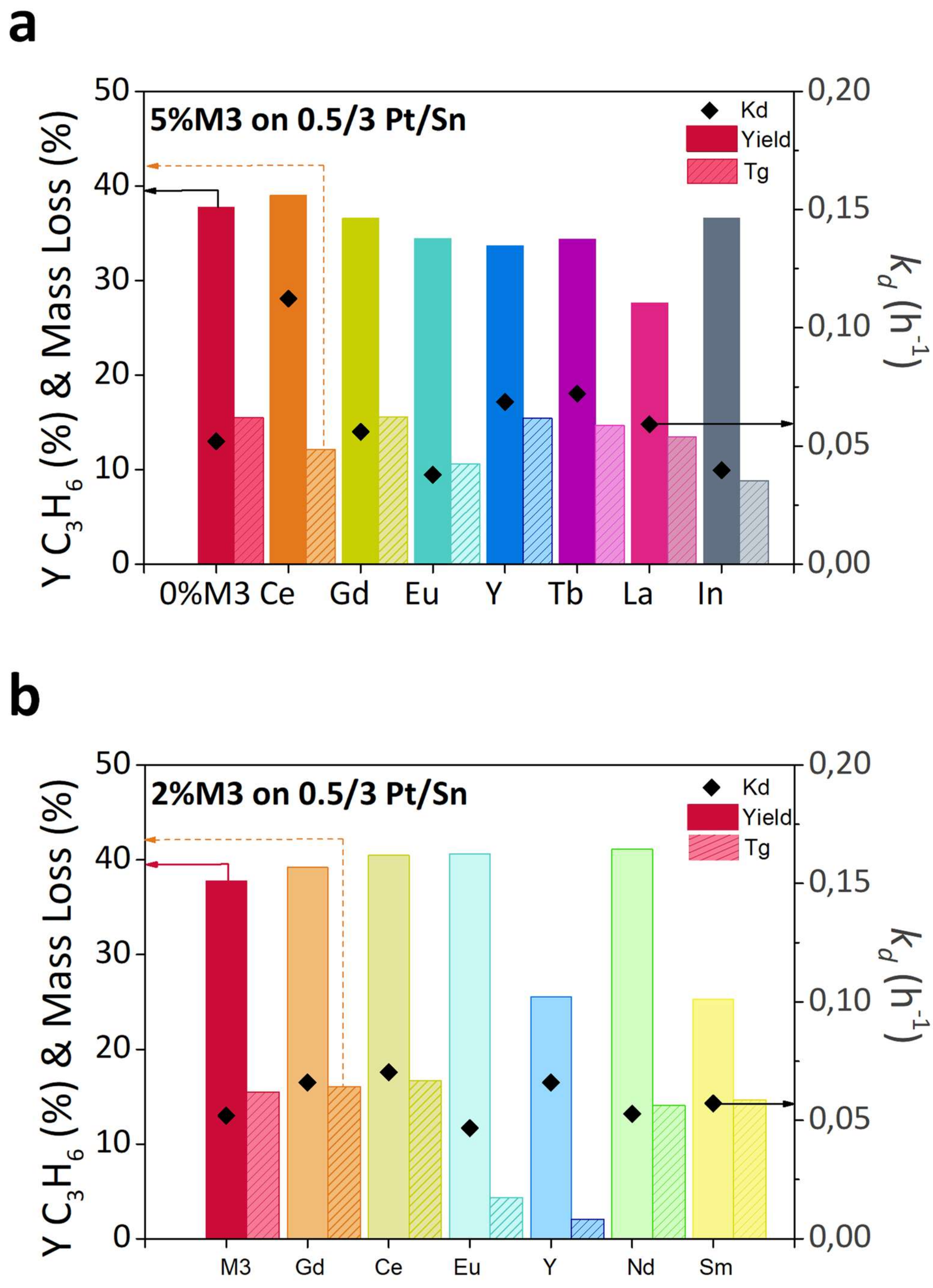

Figure S5. C_3H_6 yield (filled bars), %mass loss (TG) (mesh bars), and deactivation constant (k_d) (diamond) at 575 °C and WHSV=1.6 h^{-1} C_3H_8:N_2=12:3 mL·min^{-1}. a) 5% M3-promoted Pt/Sn catalysts. b) 2% M3-promoted Pt/Sn catalysts

Table S4. H_2 volume consumption and peak temperatures from TPR.

Sample	H_2 consumption (*mL/g*)	Peak 1 position (*°C*)	Peak 2 position (*°C*)
Pt/Sn	11,09	244,0	-
5% Gd-Pt/Sn	11,46	364,4	-
2% Eu-Pt/Sn	15,50	227,0	458,3
5%Eu-Pt/Sn	16,41	238,3	460,0
5% Ce-Pt/Sn	19,34	219,2	-

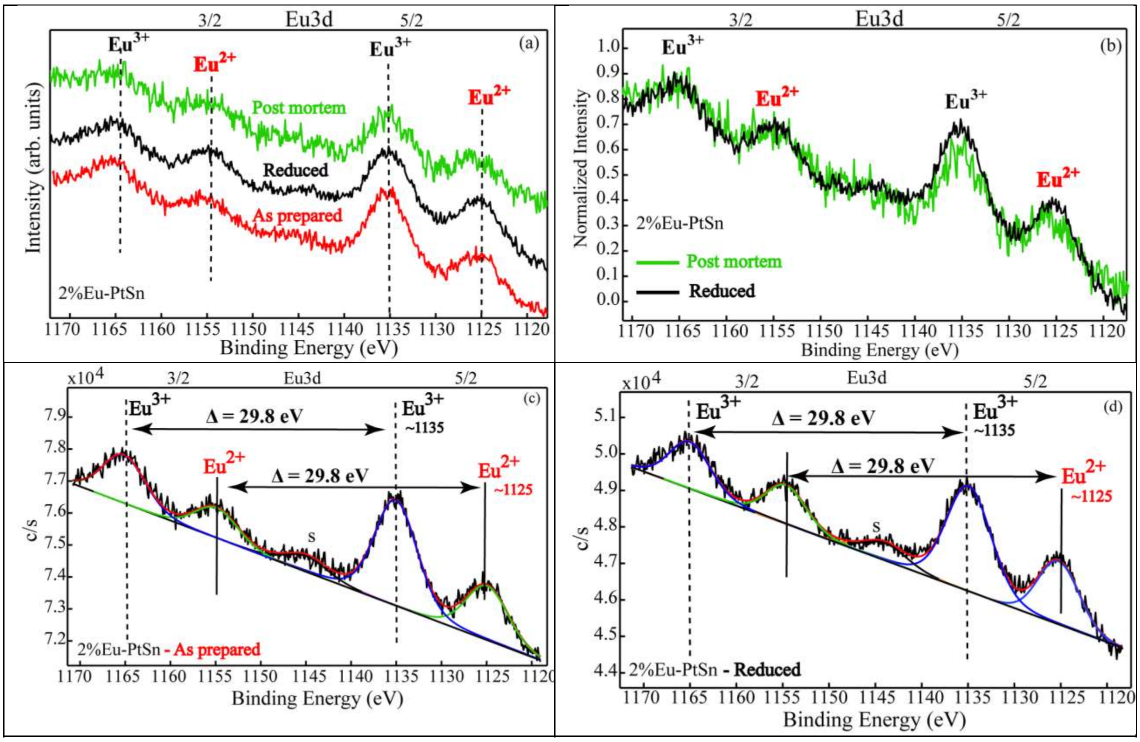

Figure S6. The superimposed Eu3d (a) for 2%Eu-PtSn catalyst as prepared, after H2 reduction and post mortem; the normalized Eu3d photoelectron spectra for 2%Eu-PtSn after reduction and post mortem (b); the deconvoluted Eu3d photoelectron lines for 2%Eu-PtSn As prepared (c) and after H2 reduction (d)

Figure S6 shows the Eu3d superimposed photoelectron lines recorded for the 2%Eu-PtSn catalyst as prepared, after the H_2 reduction as well as after the propane dehydrogenation reaction. One can notice that all the photoelectron lines exhibit a similar shape indicating no significant changes regarding the Eu chemistry. Moreover, the normalized Eu3d lines presented in the Figure S6b for the catalyst reduced and post-mortem confirm the above mentioned behaviour. Eu 3d core-level emission spectra are depicted in Figure S6c and d. Thus, after peak-fitting the Eu3d envelope, both the trivalent (3d5/2 located at ~ 1135 eV and 3d3/2 at ~ 1165 eV) and divalent (3d5/2 located at ~ 1125 eV and 3d3/2 at ~ 1155 eV) were highlighted. More specifically, a small increasing of Eu^{2+} oxidation state (3d5/2 at ~ 1125 eV and 3d3/2 at ~ 1155 eV) from 32.2% in the as prepared catalyst to 33.6% and 35.1 % occurs for the reduced and post-mortem catalyst. It was found that the oxidation states ratio Eu^{3+}/Eu^{2+} = 1.97 for the reduced catalyst is very close the ratio for the catalyst after reaction. Notably, no significant changes of the Eu^{3+}/Eu^{2+} ratio for the spent catalyst proves the stability of the 2%Eu-PtSn catalyst.

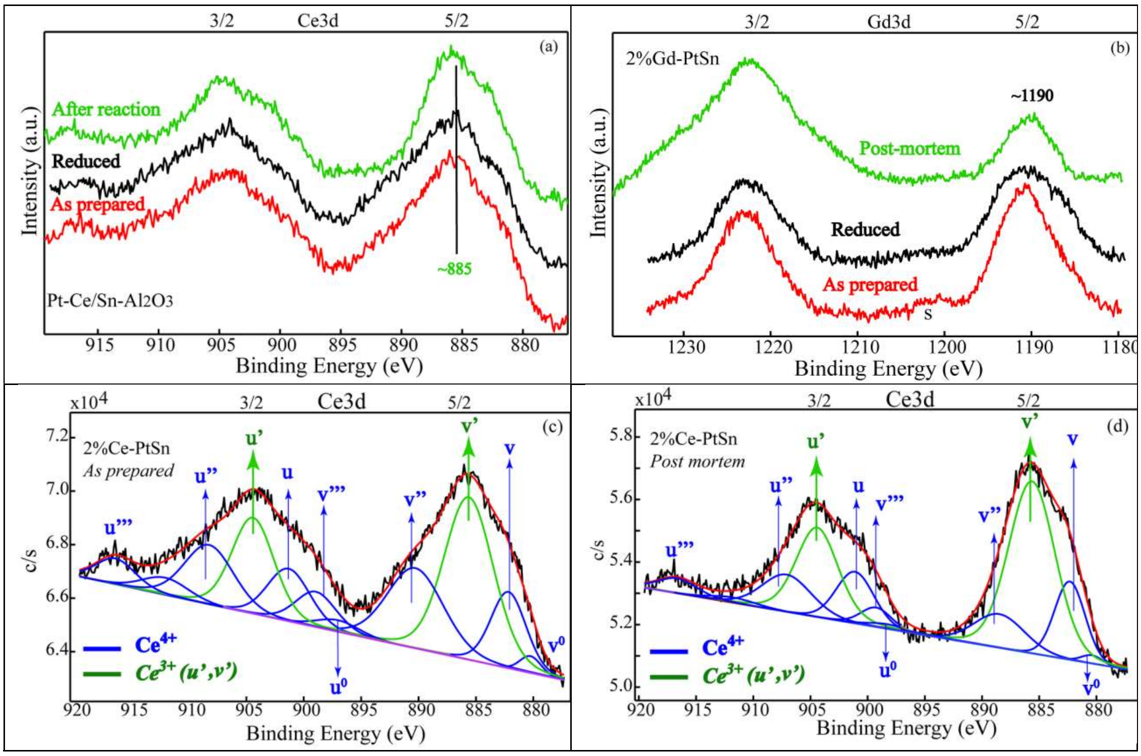

Figure S7. Ce3d (a), Gd3d (b) superimposed photoelectron spectra for 2%Ce-PtSn and 2%Gd-PtSn catalysts; the deconvoluted Ce3d photoelectron lines for 2%Ce-PtSn catalyst as prepared (c) and post-mortem (d)

Figure S7a displays the superimposed high resolution spectra of the Ce3d and for 2%Pt-SnCe catalyst in the following stages: as prepared, after H_2 reduction and after the propane dehydrogenation reaction. Ce was detected on the surface as a mixture $Ce^{4+}/Ce^{3+}$of the valence states. The Ce3d deconvoluted photoelectron broad spectra are presented in the Figure S7b and c. Next, the deconvolution procedure following Neal Fairley guidelines clearly reveals the contribution of the 3+ oxidation state. Thus, the peaks (v' and u') centered at at ~885.5 eV and at ~904.4 are assigned to Ce^{3+} valence state, while the peaks(v^0, v,v'', v''' and u^0 u,u'', u''') are attributed to the Ce^{4+} valence state. Further, the Ce^{3+} percentages were calculated from the spectral deconvolution of the Ce3d band-like spectra. 2%Ce-PtSn as prepared catalyst exhibits a fraction of Ce^{3+} around 42.6%, while for the spent catalyst this fraction increases to ~56%. The Ce chemistry for the catalyst reduced is very close to the as prepared catalyst. The explanation may be related to the oxidation of the catalyst surface after its exposure to air, although Ce^{4+} was reduced to Ce^{3+} during the H_2 treatment.

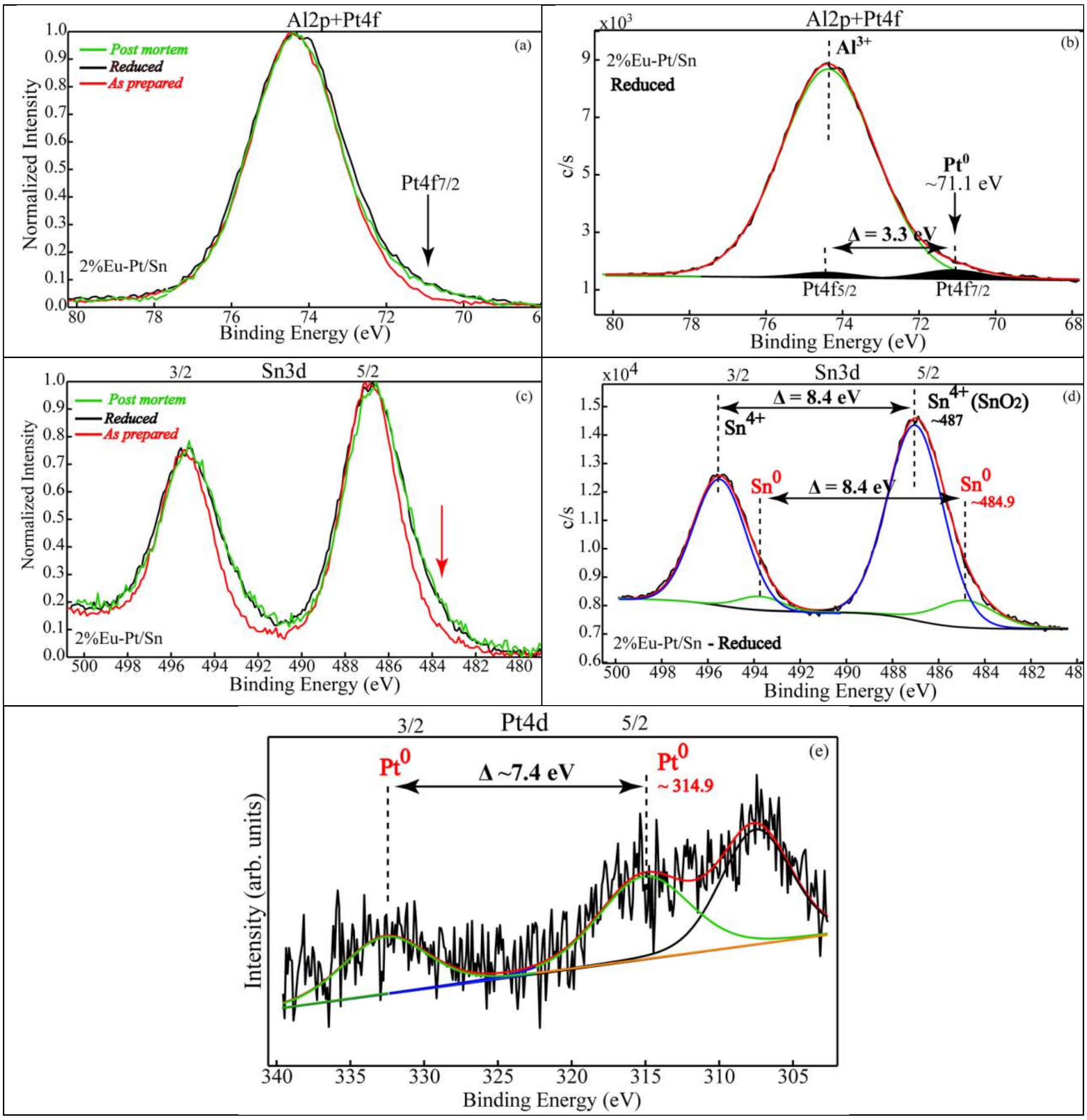

Figure S8. Al2p+Pt4f (a) and Sn3d (c) superimposed photoelectron spectra; Al2p+Pt4f (b), Sn3d (d) and Pt4d (e) deconvoluted photoelectron lines for 2%Eu – PtSn after H_2 reduction

Pt chemistry was assessed from the Pt4f and Pt4d photoelectron lines (Fig. S8a, b, and e). For the catalyst, reduced and post-mortem Pt was detected only as metallic Pt (Pt4f7/2 at ~71.1 eV and Pt4d5/2 at 314.9 eV), confirmed by the fitting curves of Al2p+Pt4f and Pt4d, respectively.

The superimposed Sn3d photoelectron lines, for the catalysts as prepared, reduced, and post-mortem (Fig. S8c), clearly reveal the reduction process of Sn. Thus, while on the surface of the 2%Ce-PtSn catalyst, Sn was found fully oxidized, on the surface of the reduced and spent catalyst, we found metallic Sn. The spectral fitting of Sn3d line (Fig. S8d) with the two peaks due to spin-orbit splitting (Δ = 8.4 eV) positioned at ~484.9 eV and ~487.0 eV for Sn 3d5/2 confirms the presence of the metallic Sn (Sn^0) and fully oxidized Sn^{4+} (SnO_2). Note that the metallic Sn detected on the surface could be alloyed with the metallic Pt detected on the surface, in agreement with STEM-EDS analysis. Concerning the Sn chemistry, we emphasize that no changes occurred after the reaction. Moreover, the formation of the $PtSn_2$ alloy could be possible, taking into account the Pt^0/Sn^0 ratio ~1:1.5, quantified by XPS.

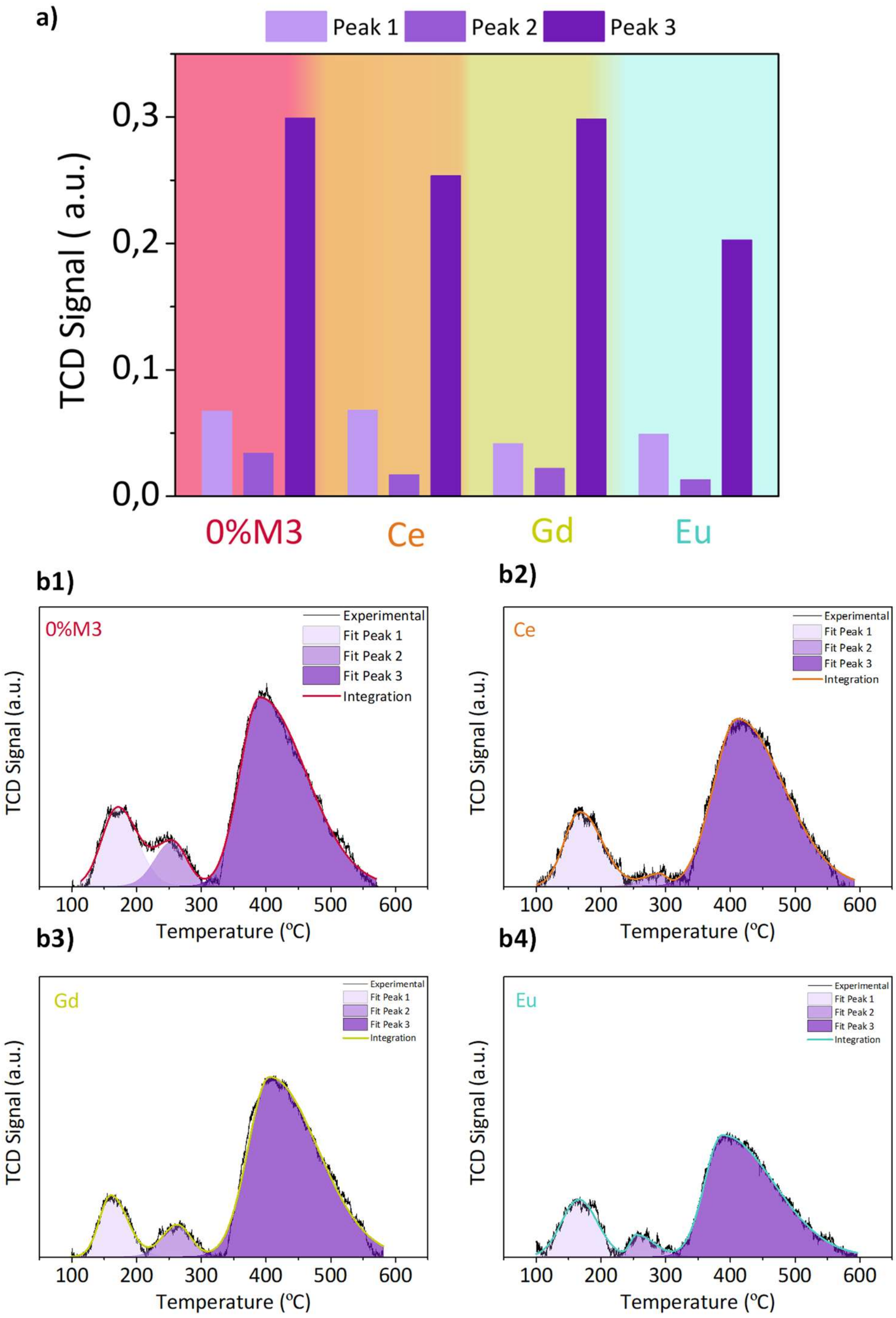

Figure S9. a) Area of NH_3 desorbed each per peak for the TPD-NH_3. b1 to b4) Deconvoluted TPD-NH_3 peaks for each catalyst. b) Deconvoluted TPD-NH_3 peaks for each catalyst. The area integration of NH_3 consumed per peak.

Table S5: Deconvoluted TPD-NH3 peaks for each catalyst. The area of NH_3 consumed per peak.

	Peak 1		Peak 2		Peak 3		Total
	**T (°C)**	**Area**	**T (°C)**	**Area**	**T (°C)**	**Area**	**Acidity**
**0%M3**	170,75	0,07	255,59	0,03	390,58	0,30	0,40
**Ce**	167,73	0,07	289,92	0,02	409,17	0,25	0,34
**Gd**	160,23	0,04	262,83	0,02	405,89	0,30	0,36
**Eu**	175,64	0,05	258,05	0,01	388,12	0,20	0,26

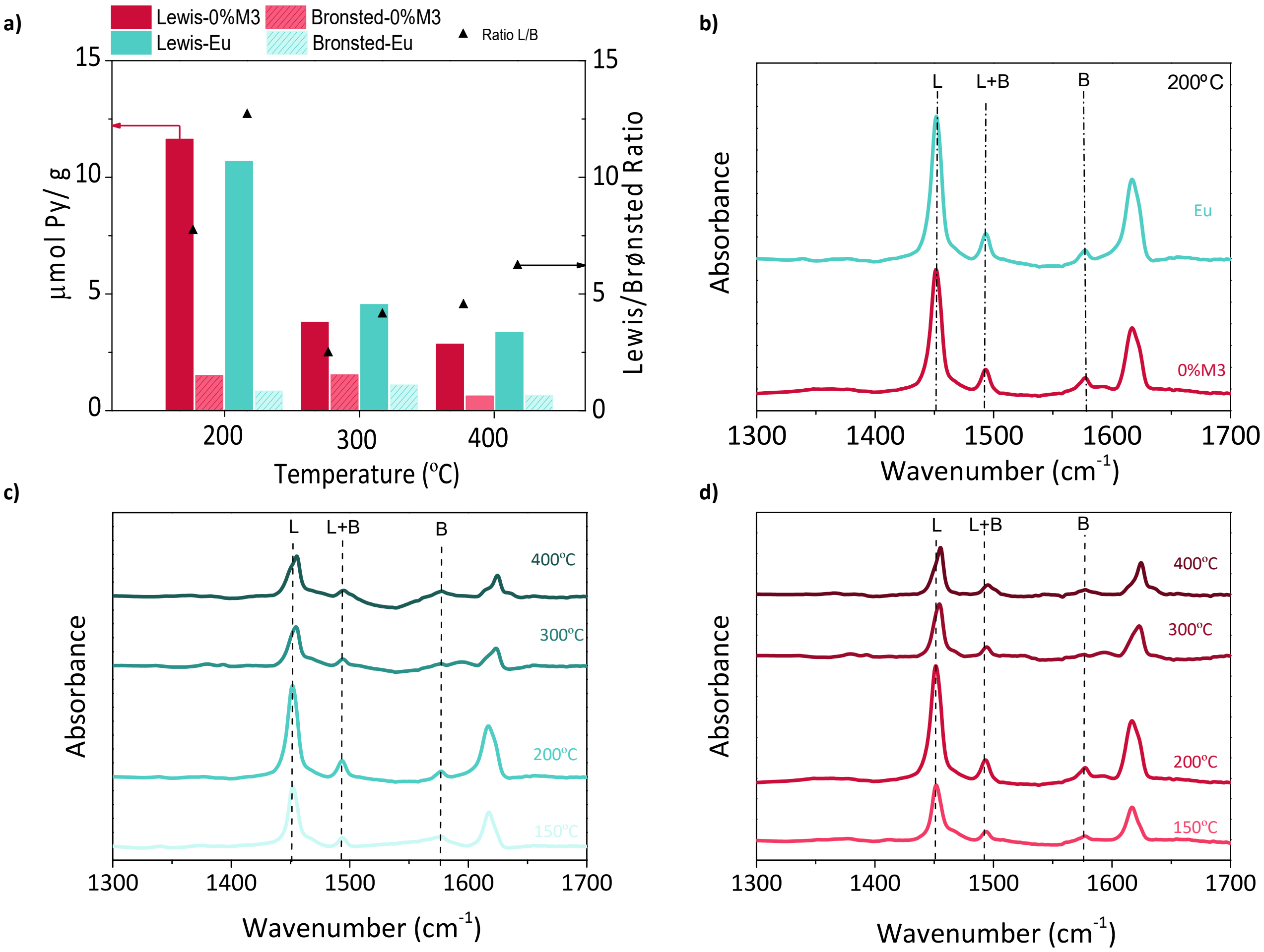

Figure S10: IR-Pyridine results for each temperature for the reference catalyst (0%M3) compared to the catalyst with Eu. a) Total acidity compared with Lewis/Brønsted ratio. Lewis or Bronsted contribution per temperature added. b) Comparison between the reference catalyst and the Eu catalyst at 200 °C. c) IR-pyridine spectra for the reference catalyst at different temperatures. d) IR-pyridine spectra for the Eu catalyst at different temperatures

Table S6: Deconvoluted IR peaks for each catalyst. The area of absorbance per peak.

	0% M3 (µmol Py/g)				Eu (µmol Py/g)			
**Wavenumber (cm^{-1})**	**150°C**	**200°C**	**300°C**	**400°C**	**150°C**	**200°C**	**300°C**	**400°C**
**L=1450**	5,36	11,63	3,79	3,29	7,00	10,68	4,55	3,35
**M=1490**	1,08	2,43	1,12	1,11	1,25	2,31	0,895	0,77
**B=1577**	5,15	1,51	1,54	0,69	1,53	0,84	1,10	0,65
**Total Acidity**	11,59	15,57	6,45	5,09	9,78	13,83	6,54	4,78
**Ratio L/B**	1,04	7,70	2,47	4,79	4,57	12,68	4,13	5,13

*NOTE: For these calculations, the extinguishing coefficients utilized are those provided by Emeis.[2]

Table S7. Thermogravimetry (TG) analysis in air of the catalysts after the reaction.

Sample	Catalyst composition	Mass loss (%)
0% M3	0.5%Pt/3%Sn/γ-Al_2O_3	15,86
2% Ce	0.5%Pt/3%Sn/2%Ce-γ-Al_2O_3	16,07
5% Ce	0.5%Pt/3%Sn/5%Ce-γ-Al_2O_3	12,12
1% Eu	0.5%Pt/3%Sn/1%Eu-γ-Al_2O_3	7,24
2% Eu	0.5%Pt/3%Sn/2%Eu-γ-Al_2O_3	4,33
3% Eu	0.5%Pt/3%Sn/3%Eu-γ-Al_2O_3	6,75
4% Eu	0.5%Pt/3%Sn/4%Eu-γ-Al_2O_3	8,9
5% Eu	0.5%Pt/3%Sn/5%Eu-γ-Al_2O_3	10,63
5% Tb	0.5%Pt/3%Sn/5%Tb-γ-Al_2O_3	
5% Y	0.5%Pt/3%Sn/5%Y-γ-Al_2O_3	15,43
5% La	0.5%Pt/3%Sn/5%La-γ-Al_2O_3	13,45
5% In	0.5%Pt/3%Sn/5%In-γ-Al_2O_3	8,83
5% Gd	0.5%Pt/3%Sn/5%Gd-γ-Al_2O_3	15,59
2% Gd	0.5%Pt/3%Sn/2%Gd-γ-Al_2O_3	16,71
2% Nd	0.5%Pt/3%Sn/2%Nd-γ-Al_2O_3	14,1
2% Y	0.5%Pt/3%Sn/2%Y-γ-Al_2O_3	
2% Sm	0.5%Pt/3%Sn/2%Sm-γ-Al_2O_3	

*Assuming water release between 0-200°C

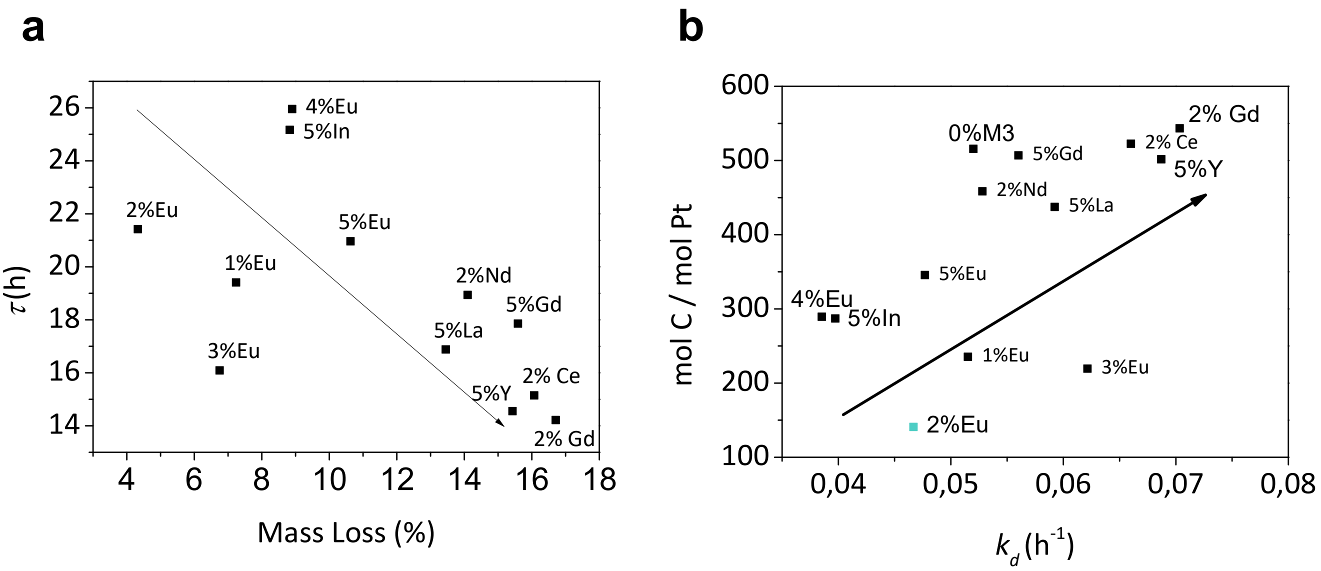

Figure S11. a) Catalyst life versus TG mass loss. b) TG-analysis: mol C consumed per mol Pt used of the spent catalysts as a function of the deactivation constant.

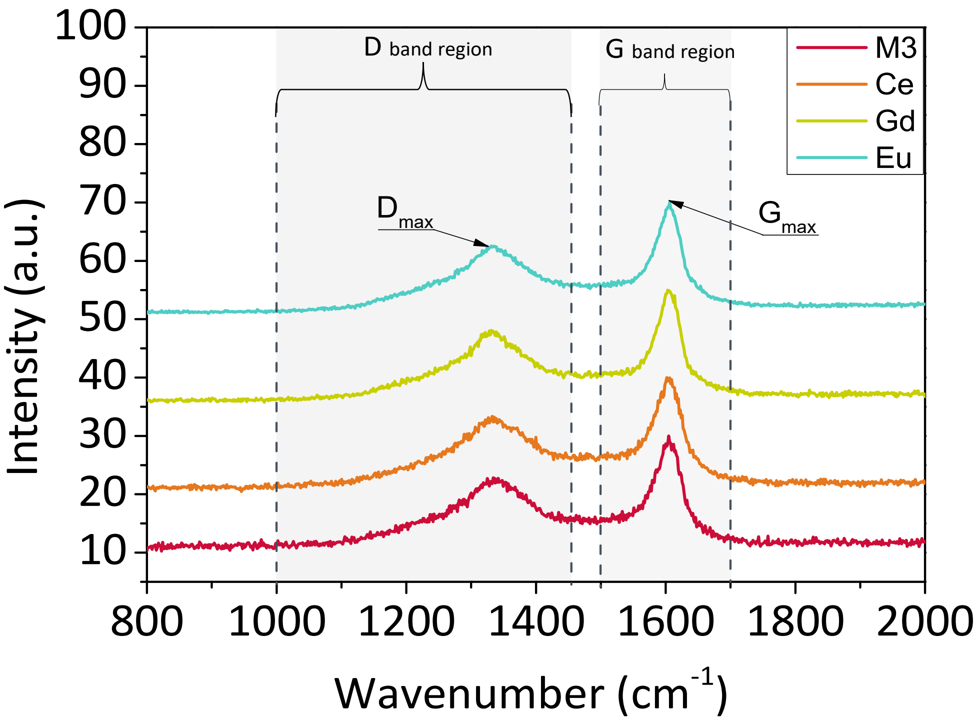

Figure S12. Raman spectra of post-mortem Ce, Gd, Eu catalysts.

Table S8: Raman results for each catalyst.

Sample	ω_D (cm^{-1})	ω_G (cm^{-1})	D band area (a.u.)	G G band area(a.u.)	D band intensity (a.u.)	G band intensity (a.u.)	I_D/I_G
0% M3	1334,1	1604,7	1697,8	1194,5	10,5	17,7	0,59
Ce	1332,6	1601,7	1962,9	1414,0	11,4	18,0	0,63
Gd	1331,1	1603,2	1577,4	1435,9	11,6	18,5	0,63
Eu	1331,2	1606,3	1596,8	1230,9	10,0	17,5	0,57

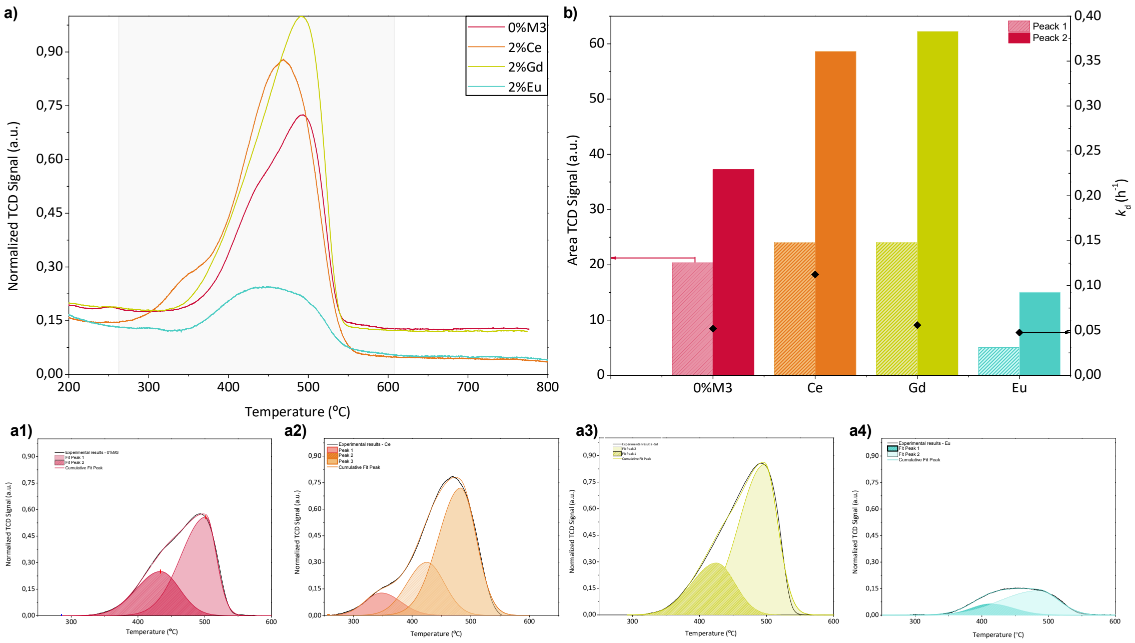

Figure S13. a) Temperature-Programmed Oxidation (TPO) after the reaction. a1 to a4) Deconvoluted TPO peaks for each catalyst. b) The area of O_2 consumed per peak compared with the deactivation constant.

Table S9: Deconvoluted TPO peaks for each catalyst. The area of O_2 consumed per peak compared with deactivation constant.

	**Peak 1**		**Peak 2**		**Global peak**				
**Sample**	**Area (a.u.)**	**T (°C)**	**Area (a.u.)**	**T (°C)**	**Area (a.u.)**	**T (°C)**	k_d	***Peak1/ Total Area (%)***	***Peak2/ Total area (%)***
0% M3	20,41	433,7	37,25	501,6	57,65	492,9	0,052	35,38	64,62
Ce	23,99	424,8	58,61	482,5	82,60	471,6	0,111	29,05	70,95
Gd	24,00	425,1	62,24	496,9	86,24	490,5	0,056	27,83	72,17
Eu	5,04	412,5	15,05	484,6	20,09	443,6	0,047	25,09	74,91

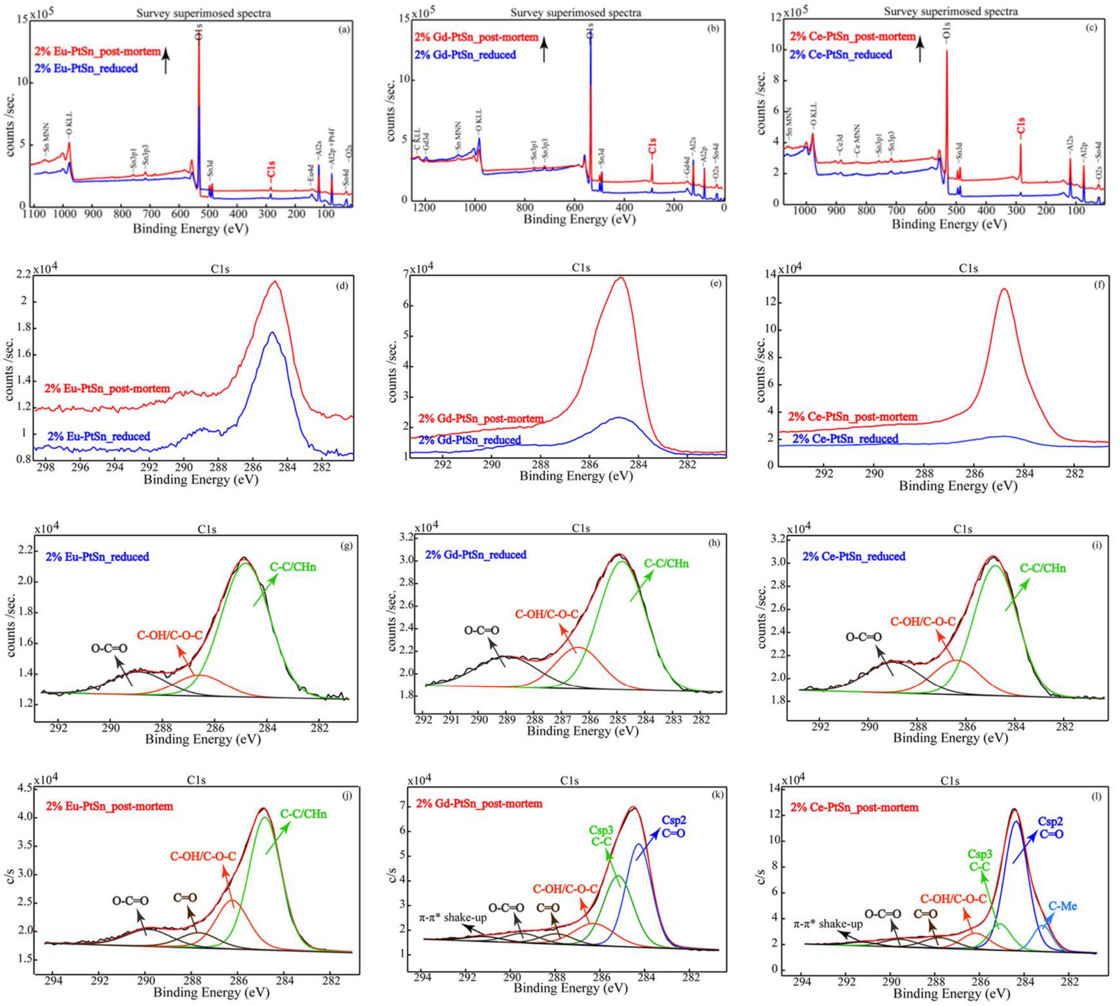

Figure S14. Survey superimposed XPS spectra for the 2%Eu (a), Gd (b), Ce (c)-PtSn catalysts after H2 reduction and post-mortem; superimposed C1s high resolution spectra for 2%Eu, Gd, Ce-Pt-Sn catalysts reduced and after reaction (d-f); C1s deconvoluted spectra for 2%Eu, Gd, Ce-Pt-Sn after H_2 reduction (g-i) and post-mortem (j-l)

Just an inspection at a glance of the superimposed survey spectra for the reduced and post-mortem catalysts clearly sheds light on a significant increase in the carbon content only on the surfaces of the 2%Gd-PtSn and 2%Ce-PtSn post-mortem catalysts. While the amount of carbon on the surfaces of all reduced catalysts is around 5 wt.%, the surfaces of the post-mortem catalysts exhibit an increased carbon content up to ~14 wt.% for 2%Gd-PtSn and 22 wt.% for 2%Ce-PtSn, respectively (Fig. S12a-f). Clearly, the addition of the carbon for 2%Eu-PtSn catalyst during propane dehydrogenation reaction catalytic is achieved at a ultra low rate (Fig. S12a, d). The carbon amount after reaction is very close to the value of carbon for the reduced 2%Eu-PtSn catalyst. The carbon chemical species were highlighted for all reduced and postmortem catalysts, after peak-fitting the C1s envelope (Fig. S12g-l). Thus, the sp2 and sp3 hybridized carbon chemical species located at 284.5 and 285.0 eV were detected only on the surfaces of 2%Gd-PtSn and 2%Ce-PtSn catalysts, in good agreement with Raman results. Moreover, the presence of the small peak at a binding energy of 291.5 eV, attributed to the π-π shake-up transition, is a characteristic feature of graphitic carbon (C=C). No graphitic carbon was detected on the surface, despite its evidence by Raman spectroscopy. This finding could be related to the segregation/diffusion processes that occurred between bulk and surface.

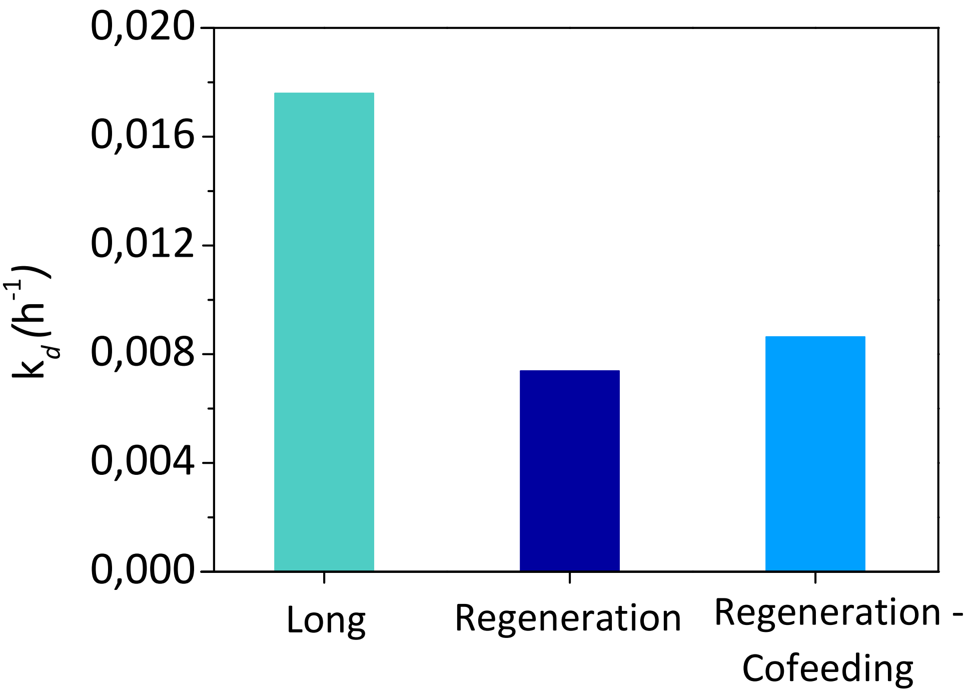

Figure S15: Deactivation constant (k_d) for Long-term PDH reaction for 150 h using a 0.5%Pt-3%Sn-2%Eu γ-Al_2O_3 catalyst at 575 °C, WHSV=1.6 h^{-1}, and a C_3H_8:N_2 ratio of 12:3 mL·min^{-1}, shown in light green, versus regeneration cycles, dark blue and Co-feeding regeneration cycles light blue. Each cycle includes 10.6 h of reaction (under the same conditions or H_2:C_3H_8 = 0.5 respectively), 4 h of regeneration with air at 50 mL/min, and 2 h of reactivation with H_2: Ar at a 1:1 ratio.

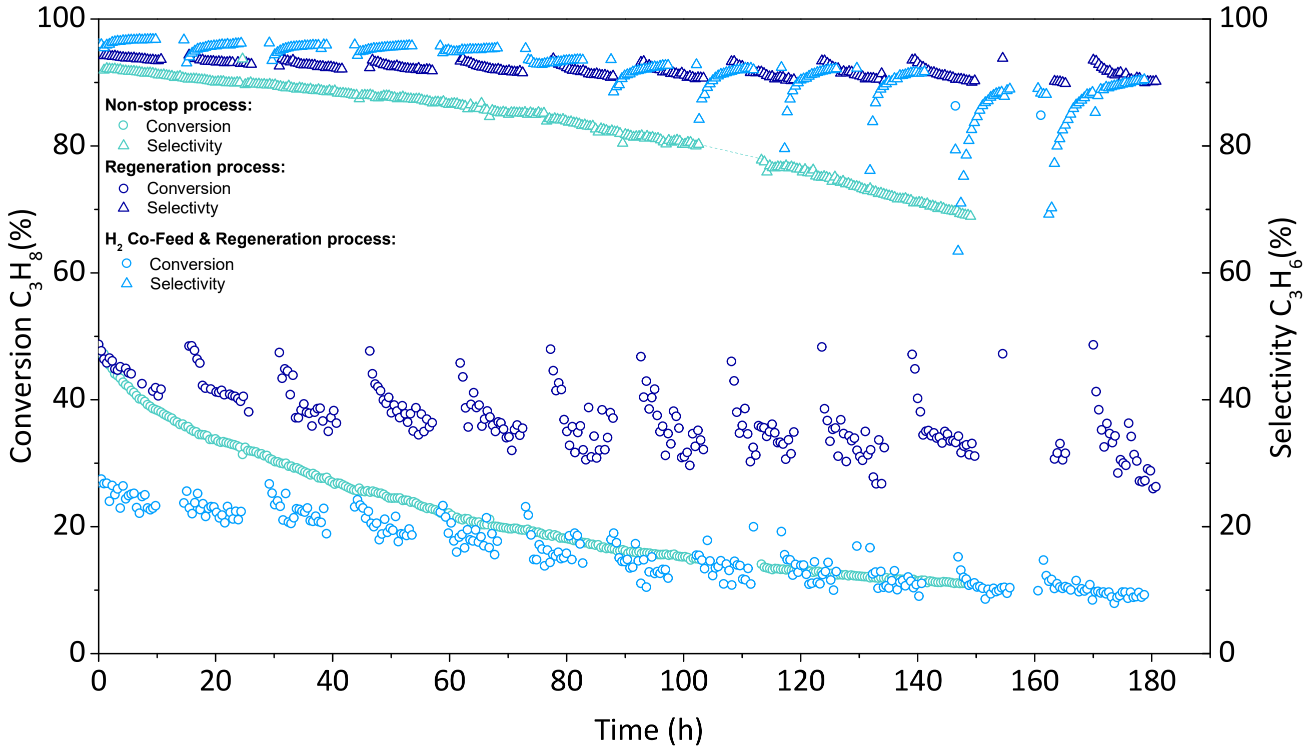

Figure S16. Long-term PDH reaction for 150 h using a 0.5%Pt-3%Sn-2%Eu γ-Al2O$_3$ catalyst at 575 °C, WHSV=1.6 h^{-1}, and a C_3H_8:N_2 ratio of 12:3 mL·min^{-1}, shown in light green, versus regeneration cycles, dark blue, and Co-feeding regeneration cycles, light blue. Each cycle includes 10.6 h of reaction (under the same conditions or H_2:C_3H_8 = 0.5, respectively), 4 h of regeneration with air at 50 mL/min, and 2 h of reactivation with H_2:Ar at a 1:1 ratio.

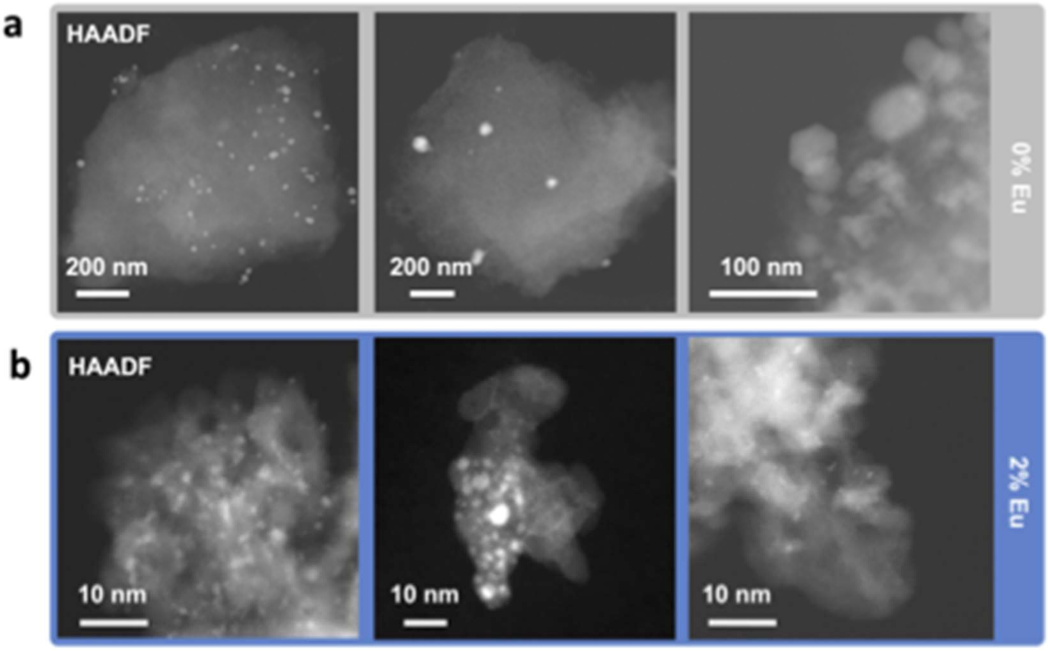

Figure S17. Overview HAADF STEM images of the non (a) and 2% Eu promoted (b) Pt-Sn catalyst.

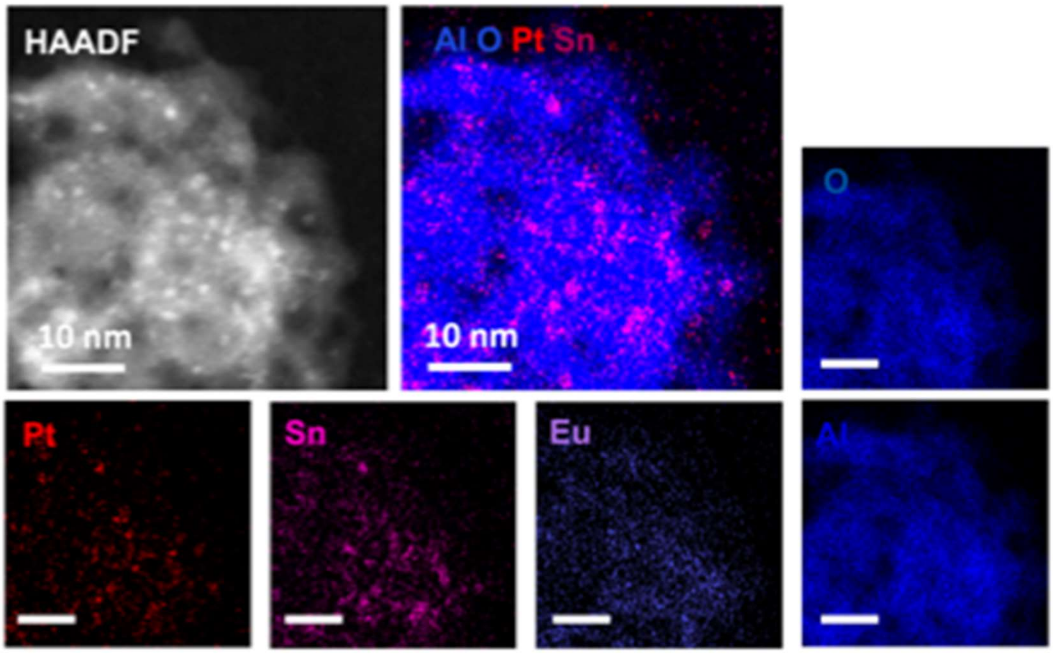

Figure S18. Overview STEM EDS mappings of the 2% Eu-promoted Pt-Sn catalyst. All scale bars in EDS mappings are 10 nm.

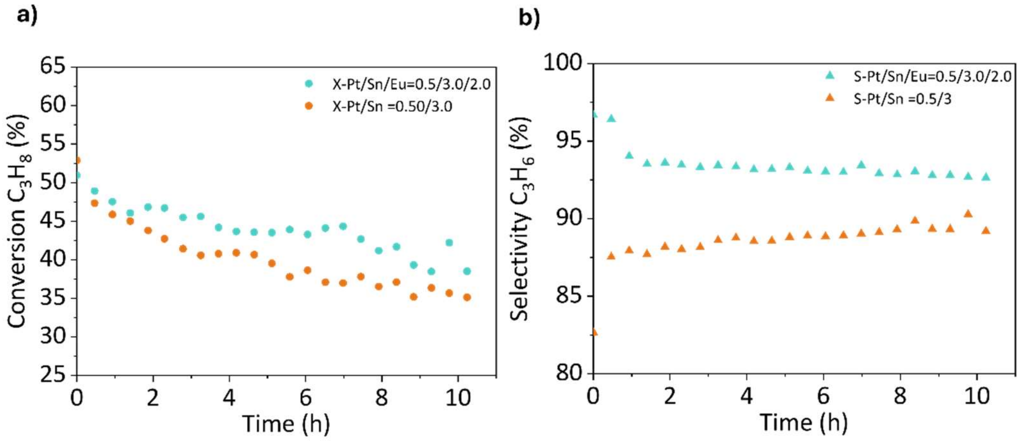

Figure S19: Conversion of C_3H_8 (a, circles) and coke-free selectivity to C_3H_8 (b, triangles) vs time on stream at 575 °C, WHSV = 1.6 h^{-1}, C_3H_8:N_2 = 80:20. Comparison between with or without Eu in the synthesis. Ratio expressed in atomic form based on ICP results

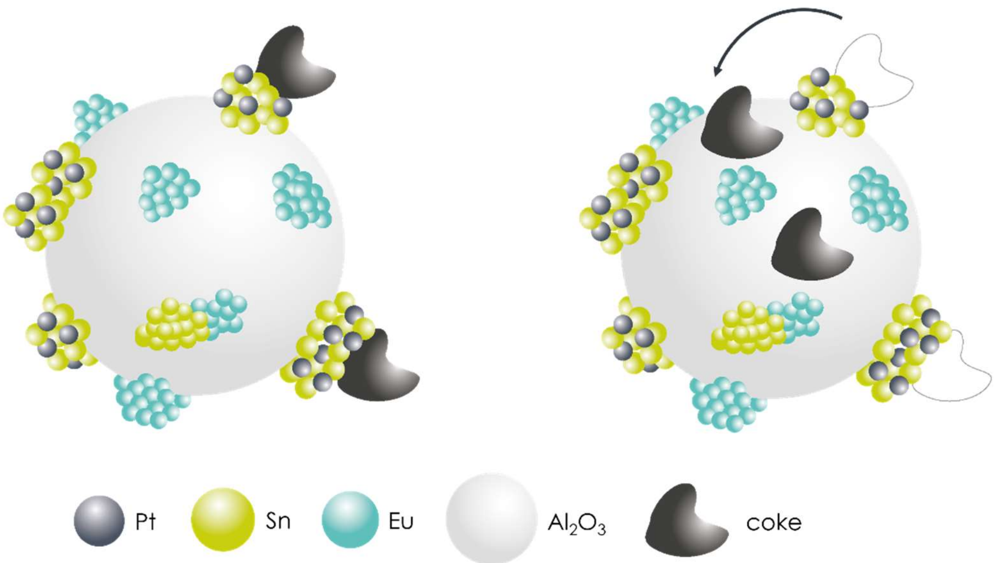

Figure S20: Schematic illustration of Pt-Sn (1:2) nanoparticles promoted with Eu. Eu facilitates coke delocalization from Pt sites to the surface, thereby mitigating Pt deactivation.

References

[1] Y. Nakaya, F. Xing, H. Ham, K. Shimizu, S. Furukawa, *Angewandte Chemie International Edition* **2021**, *60*, 19715–19719.

[2] J. Zhu, R. Osuga, R. Ishikawa, N. Shibata, Y. Ikuhara, J. N. Kondo, M. Ogura, J. Yu, T. Wakihara, Z. Liu, T. Okubo, *Angewandte Chemie International Edition* **2020**, *59*, 19669–19674.

[3] X. Fan, J. Li, Z. Zhao, Y. Wei, J. Liu, A. Duan, G. Jiang, *Catal Sci Technol* **2015**, *5*, 339–350.

[4] L. Liu, M. Lopez-Haro, C. W. Lopes, S. Rojas-Buzo, P. Concepcion, R. Manzorro, L. Simonelli, A. Sattler, P. Serna, J. J. Calvino, A. Corma, *Nat Catal* **2020**, *3*, 628–638.

[5] K. Searles, K. W. Chan, J. A. Mendes Burak, D. Zemlyanov, O. Safonova, C. Copéret, *J Am Chem Soc* **2018**, *140*, 11674–11679.

[6] H. N. Pham, J. J. H. B. Sattler, B. M. Weckhuysen, A. K. Datye, *ACS Catal* **2016**, *6*, 2257–2264.

[7] Y. Xu, J. Chen, X. Yuan, Y. Zhang, J. Yu, H. Liu, M. Cao, X. Fan, H. Lin, Q. Zhang, *Ind Eng Chem Res* **2018**, *57*, 13087–13093.

[8] M.-H. Lee, B. M. Nagaraja, K. Y. Lee, K.-D. Jung, *Catal Today* **2014**, *232*, 53–62.

[9] Y. Zhang, Y. Zhou, J. Shi, S. Zhou, Z. Zhang, S. Zhang, M. Guo, *Fuel Processing Technology* **2013**, *111*, 94–104.

[10] B. K. Vu, M. B. Song, I. Y. Ahn, Y. W. Suh, D. J. Suh, W. Il Kim, H. L. Koh, Y. G. Choi, E. W. Shin, *Catal Today* **2011**, *164*, 214–220.

[11] Y. Zhang, Y. Zhou, J. Shi, S. Zhou, X. Sheng, Z. Zhang, S. Xiang, *J Mol Catal A Chem* **2014**, *381*, 138–147.

[12] J. Im, M. Choi, *ACS Catal* **2016**, *6*, 2819–2826.

[13] Y. Qiu, X. Li, Y. Zhang, C. Xie, S. Zhou, R. Wang, S. Z. Luo, F. Jing, W. Chu, *Ind Eng Chem Res* **2019**, DOI 10.1021/acs.iecr.9b01413.

[14] L. Shi, G.-M. Deng, W.-C. Li, S. Miao, Q.-N. Wang, W.-P. Zhang, A.-H. Lu, *Angewandte Chemie International Edition* **2015**, *54*, 13994–13998.

[15] B. Li, Z. Xu, F. Jing, S. Luo, W. Chu, *Appl Catal A Gen* **2017**, *533*, 17–27.

[16] R. Ryoo, J. Kim, C. Jo, S. W. Han, J. C. Kim, H. Park, J. Han, H. S. Shin, J. W. Shin, *Nature* **2020**, *585*, 221–224.
